# The dimeric structure of wild-type human glycosyltransferase B4GalT1

**DOI:** 10.1371/journal.pone.0205571

**Published:** 2018-10-23

**Authors:** Deborah Harrus, Fawzi Khoder-Agha, Miika Peltoniemi, Antti Hassinen, Lloyd Ruddock, Sakari Kellokumpu, Tuomo Glumoff

**Affiliations:** Faculty of Biochemistry and Molecular Medicine, University of Oulu, Aapistie 7A, Oulu, Finland; University of Toulouse - Laboratoire d'Ingénierie des Systèmes Biologiques et des Procédés, FRANCE

## Abstract

Most glycosyltransferases, including B4GalT1 (EC 2.4.1.38), are known to assemble into enzyme homomers and functionally relevant heteromers *in vivo*. However, it remains unclear why and how these enzymes interact at the molecular/atomic level. Here, we solved the crystal structure of the wild-type human B4GalT1 homodimer. We also show that B4GalT1 exists in a dynamic equilibrium between monomer and dimer, since a purified monomer reappears as a mixture of both and as we obtained crystal forms of the monomer and dimer assemblies in the same crystallization conditions. These two crystal forms revealed the unliganded B4GalT1 in both the open and the closed conformation of the Trp loop and the lid regions, responsible for donor and acceptor substrate binding, respectively. The present structures also show the lid region in full in an open conformation, as well as a new conformation for the GlcNAc acceptor loop (residues 272–288). The physiological relevance of the homodimer in the crystal was validated by targeted mutagenesis studies coupled with FRET assays. These showed that changing key catalytic amino acids impaired homomer formation *in vivo*. The wild-type human B4GalT1 structure also explains why the variant proteins used for crystallization in earlier studies failed to reveal the homodimers described in this study.

## Introduction

Beta-1,4-galactosyltransferase 1 (B4GalT1; EC 2.4.1.38) is a family 7 glycosyltransferase residing in the trans Golgi. In the presence of manganese, it transfers a galactose moiety (Gal) from UDP-galactose (UDP-Gal) to an N-acetylglucosamine (GlcNAc) residue, with the formation of a β1–4 linkage [1–4]. B4GalT1 can interact with α-lactalbumin to form the lactose synthase complex (EC 2.4.1.22), in which case its sugar acceptor specificity is modified and the acceptor can be a single glucose residue (Glc) instead of GlcNAc, resulting in the synthesis of the disaccharide lactose [5, 6]. B4GalT1 possesses a type II membrane protein topology, with a short N-terminal cytoplasmic tail and an α-helical transmembrane domain responsible for Golgi localization, a stem domain (amino acids 45–125), that is believed to be mostly disordered and whose function is still unknown, and a globular catalytic domain (amino acids 126–398) carrying the sugar transfer activity.

Previous structure-function studies of the catalytic domain of B4GalT1 by x-ray crystallography revealed that the enzyme undergoes conformational changes upon substrate binding and during its catalytic cycle. At least two regions are involved: The first one is a short loop (hereby called Trp loop, also referred to as “short loop” or “loop2” in the literature) comprised of residues 309–318 and includes Trp310, which is responsible for donor sugar specificity [7]. The Trp loop sequence is strictly conserved among the GalT protein family (B4GalT1-7), as well as among orthologues from animals. The second region is a long loop (hereby called lid, also referred to as “long loop” or “loop3” in the literature) comprising residues 338–365, whose sequence is less conserved. This loop controls the so-called open and closed conformations of the unliganded enzyme and substrate-bound enzyme, respectively. Amino acid residues 354–361 at the C-terminal end of the lid are subject to a change in their secondary structure, transitioning from random coil to α-helix between the open and closed conformation. The newly formed helix interacts with the acceptor sugar molecule. After the transfer of Gal to GlcNAc is complete, the lid must revert to the open conformation in order to release the remaining UDP moiety [6]. The lid is highly flexible in the absence of substrates and often cannot be observed in crystal structures. A third loop (hereby called loop1) comprising residues 272–288 participates in the catalytic activity. It binds the acceptor GlcNAc through hydrophobic contacts via the highly conserved Phe276 and Tyr282 [8, 9]. Loop1 has been identified as flexible by molecular dynamics simulations [10], but has always been observed in the same position in all published crystal structures of bovine or human B4GalT1.

Trp310 (Trp314 in the bovine enzyme) has been shown to play a crucial role in the conformational state of the lid, in the binding of both donor and acceptor substrates and in the catalytic mechanism of the enzyme [6, 10, 11]. In the unliganded enzyme structure, the aromatic side chain of Trp310 is exposed to the solvent [12, 13]. Upon binding of substrate Trp310 flips inside the catalytic pocket, its NE1 atom interacting with the anionic phosphate oxygen atom of UDP-Gal [14] and it anchors the acceptor oligosaccharide through hydrophobic interactions [13].

B4GalT1 is known to form high molecular weight oligomers and homodimers. This behavior has been observed for protein isolated from membrane preparations from mammalian cells [15, 16], for the purified lactose synthase complex from mammary glands, milk and colostrum [17, 18] and from human plasma [19]. More recently, the existence of homomers in live cells has been verified by utilizing bimolecular fluorescent complementation and fluorescence resonance energy transfer (FRET) in COS-7 cells [20–22]. In addition, size-exclusion chromatography confirmed that B4GalT1 exists mostly as complexes (homomers or heteromers) and not as monomers in live cells [21].

Here we report that the catalytic domain of human B4GalT1 purified from soluble cell lysates forms homodimers *in vitro*. In addition, we present two structures of the enzyme, which contain novel and interesting structural details, in the monomeric and dimeric state.

## Materials & methods

### Protein purification

The Golgi lumen resident globular catalytic domain of human B4GalT1 (residue range Asp99-Ser398) (S1 Fig) was expressed in LB medium in a BL21(DE3) *Escherichia coli* strain (Invitrogen), using a polycistronic Ptac vector containing the CyDisCo system [23] enabling disulfide bond formation in the cytoplasm of *E*. *coli*. Protein expression was induced overnight at 30°C with 1 mM Isopropyl-β-D-thiogalactopyranoside (IPTG) after the culture in 37°C had reached OD 0.6. Bacteria were pelleted (6000 x g, 20 min) and lysed by sonication in 50 mM sodium phosphate, pH 7.2. Proteins were purified with a Bio-Scale Mini Profinity Nickel cartridge (Bio-Rad) followed by a 24 ml Superdex 200 HR 10/30 column (GE Healthcare), which had been calibrated with γ-globulin (158 kDa), ovalbumin (44 kDa) and myoglobin (17 kDa). The protein was then concentrated into the final buffer (20 mM sodium phosphate, pH 7.2, 150 mM NaCl) using a 10 kDa cut-off centrifugal concentrator (Millipore). The purity was assessed to be >95% by SDS-PAGE with coomassie-blue staining.

### Enzyme activity measurements

Enzyme activity measurements were conducted using the UDP-Glo Glycosyltransferase assay kit (Promega) according to the kit manual. Prior to the experiment, B4GalT1 was dialyzed against a buffer containing 20 mM Tris-HCl and 150 mM KCl at pH 7.0. The reaction buffer used for the assay was 50 mM Bis-Tris, 5 mM MnCl_2_ pH 6.3. For the assay, a standard amount of 80 ng of B4GalT1 was used, with UDP-galactose (Promega) and ovalbumin (Sigma-Aldrich) as the donor and the acceptor, respectively. The donor was serially diluted 11 times with the highest concentration being 4 mM, while the acceptor concentration was kept constant at 3 mM. Measurements were done in triplicate after 1-hour incubation at 37°C. The reaction was stopped by the addition of the UDP detection reagent. The luminescence values of the samples were measured with a Tecan Infinite M1000 Pro luminometer.

### Crystallization and data collection

Crystals were obtained by the hanging drop vapor diffusion method at room temperature. Equal volumes of protein solution (8.6 mg/ml in 20 mM sodium phosphate, pH 7.2, 150 mM NaCl) and well solution (0.2 M potassium nitrate, 20% (w/v) PEG 3350) were mixed. Crystals were harvested and soaked for a few seconds in a cryo-solution (crystallization solution supplemented with 20% glycerol) and flash-cooled in liquid nitrogen. Two high-resolution synchrotron datasets (Table 1) were collected remotely at the beamline i04 of the Diamond Light Source (Oxfordshire, UK).

**Table 1:** Crystallographic data collection and refinement statistics. Statistics for the highest-resolution shell are shown in parentheses.

	Orthorhombic (6FWT)	Trigonal (6FWU)
Space group	P 2_1_ 2_1_ 2	P 3_1_ 2 1
Cell parameters (Å, °)	42.3 43.7 144.3 90 90 90	60.2 60.2 229.4 90 90 120
Resolution range (Å)	42.28–1.85 (1.91–1.85)	47.47–2.35 (2.43–2.35)
Molecules per asymmetric unit	1	2
V_m_ (Å^3^/D)	1.835	1.651
Mean I/sigma (I)	11.47 (1.16)	10.48 (1.78)
Completeness (%)	99.8 (99.2)	99.8 (99.5)
Unique reflections	23816 (2316)	21046 (2045)
Multiplicity	6.0 (3.8)	9.3 (9.5)
RMSD bonds(Å)/angles (°)	0.012/1.07	0.016/1.27
R-work/ R-free (%)	16.63/21.69 (26.67/31.23)	18.28/25.80 (25.75/32.12)
Wilson B-factor	28.3	37.3
Number of atoms (non-H)	2481	4056
protein	2280	3998
water	195	50
glycerol	6	-
nitrate	-	8
Ramachandran favoured (%)	97.8	97.0
Ramachandran allowed (%)	2.2	3.0

### Crystal structure determination, refinement and visualization

Data were processed with the XDS package [24, 25]. The previously published crystal structure of human B4GalT1 (Protein Data Bank (PDB) code 4EE3) truncated of the flexible loops (residues 273–285, 309–326 and 340–365) was used as the search model for molecular replacement. Molecular replacement was performed with Phaser [26]. Models were rebuilt with COOT [27] and refined with phenix.refine [28]. Structural figures were generated using the PyMOL Molecular Graphics System, Version 1.3 Schrödinger, LLC.

### Dimer interface analysis

The nature of the interface and thermodynamic properties were assessed employing the jsPISA macromolecular surface and interface calculation tool [29] and Voronoi tessellation, i.e. the DiMoVo server [30]. Evolutionary conservation of the interface was assessed using the InterEvol server [31]. The jsPISA score was computed as described in [32].

### Targeted mutagenesis and FRET plasmid constructs

Please refer to the procedures in the supplemental materials S1 Text.

### High Content FRET interaction measurements

COS-7 cells (ATCC, Manassas, VA; cat. no. CRL-1651) grown in 35 mm dishes were transfected with the appropriate plasmids 20 h after plating. The transfection mix contained 95 μl of serum free DMEM, 5 μl of FuGENE 6 (Promega) and 500 ng of each plasmid. Golgi localization of all of the constructs was verified first as described previously [22] using immunofluorescence microscopy and the anti-GM130 antibody as a Golgi marker (BD Biosciences). For the FRET measurements, cells were transferred to a 96-well plate (6 separate wells/transfection, each well containing 3000–5000 cells) 16–20 h after transfection and then allowed to attach for additional 4 to 6 hours before fixation with 4% paraformaldehyde and washing with PBS. FRET signals were measured using the Operetta High Content Imaging System (Perkin Elmer) and appropriate filter sets for mVenus and mCherry and FRET (mVenus, 475/520 nm; mCherry, 570/615 nm; FRET, 475/615 nm). Harmony software (Perkin Elmer) was used for quantification of the FRET signals with the Youvan correction [33]. All data are represented as FRET efficiencies (mean ± SD, n = 3) as described by Kokkonen et al. [34]. Student’s two-tailed t-test was used to calculate statistical significance, with p < 0.05 considered to be statistically significant.

### Blue native PAGE and Western blot

Protein samples were mixed with Tris/glycerol native sample buffer and loaded onto a 4–12% polyacrylamide gel. The gel was run in Tris/glycine buffer supplemented with 0.02% Coomassie Brilliant Blue G-250 dye until the samples had migrated 1 cm into the gel, then without the dye at 20 mA for 2 hours. Bovine serum albumin (BSA Fraction V, Sigma) was used as a reference for molecular weight. The samples were then transferred onto a 0.45 μm nitrocellulose membrane (Amersham Protran, GE Healthcare) for 2 hours at 200 mA in a transfer buffer (25 mM Tris, 192 mM glycine, pH 8.3). The membrane was quenched by using 5% non-fat milk in TBS-Tween (50 mM Tris, 150 mM NaCl, 0.02% Tween 20, pH 7.6) supplemented with 0.1% BSA overnight at 4°C. The blot was then incubated with Anti-B4GalT1 antibody (HPA010807, Sigma,1:2000) in TBS-Tween + 0.1% BSA for 2 hours at room temperature, washed 3 x 10 min with TBS-Tween before incubation with the goat anti-rabbit HRP 1:10000 (Abliance, Compiègne, France) in TBS-Tween + 0.1% BSA for 1 hour at room temperature. After final washings (4 x 15 min in TBS-Tween), ECL reagent (BioRad) was added and the membrane was photographed using a GelDoc instrument (BioRad). A linear correlation between the log of the molecular weight of BSA oligomers and their migration distance was used to estimate the molecular weights of oligomeric species of B4GalT1.

## Accession numbers

### Protein Data Bank accession codes

Atomic coordinates and structure factors of human wild-type B4GalT1 in the open and closed conformation have been deposited with the PDB with accession codes 6FWT and 6FWU, respectively.

## Results

### Enzymatic activity and oligomerization state of the crystallizable B4GalT1

**Fig 1:**
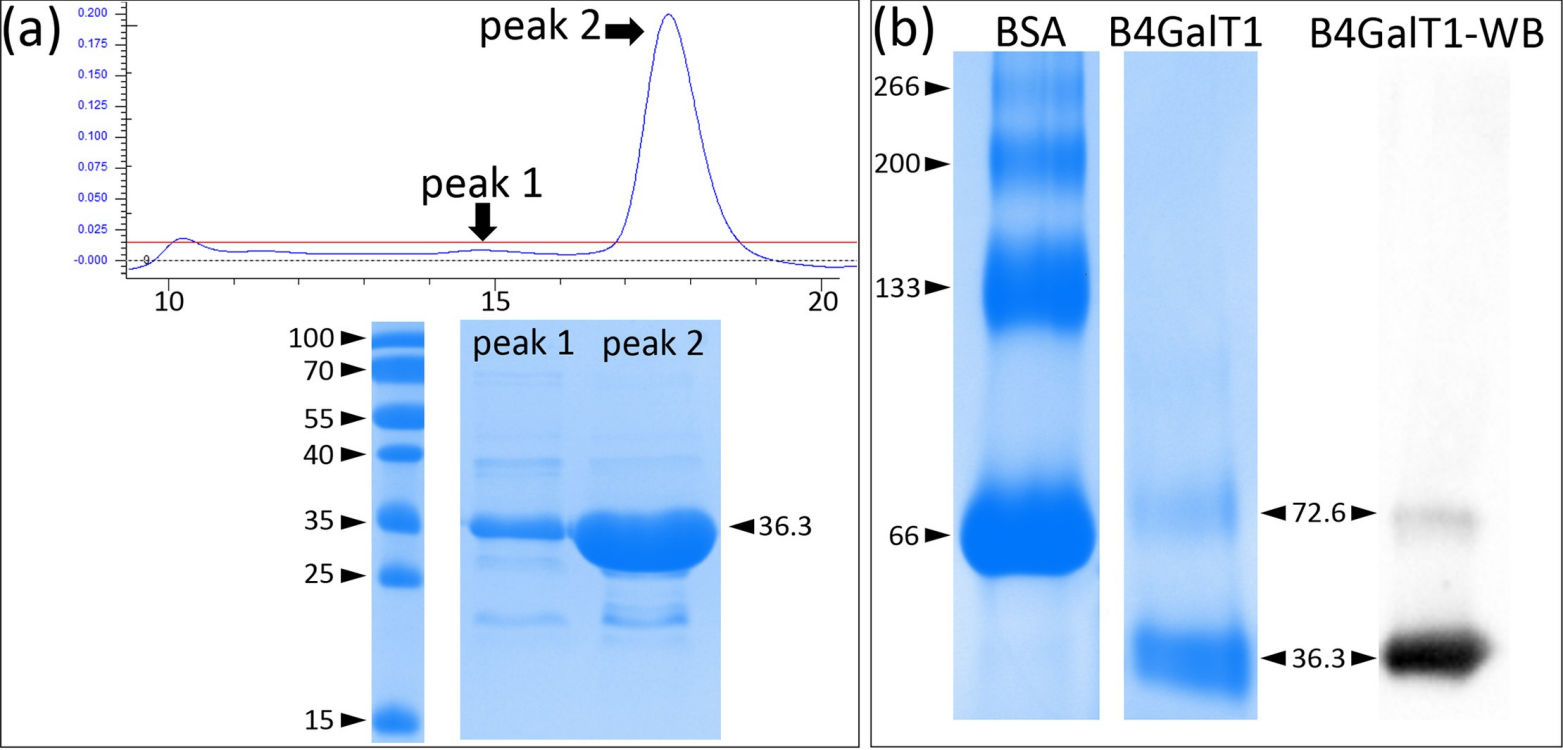
The oligomeric state of the purified B4GalT1 catalytic domain. (a). Gel filtration and SDS-PAGE profiles of the purified B4GalT1 catalytic domain. Molecular weight calculated from the sequence of the construct is 36.35 kDa. Elution volumes of the calibration proteins of the gel filtration column formed a standard curve with R^2^ value of 0.9993. Elution volumes, as seen from the scale in milliliters, are 14.7 ml for peak 1 and 17.6 ml for peak 2, and they correspond to molecular weights (indicated by arrows and figures in kDa) of 72.6 (dimer) and 36.3 kDa (monomer), respectively. PageRuler Prestained Protein Ladder (Thermo Fisher) was used as the molecular weight standard. (b). The two leftmost lanes show BSA (bovine serum albumin) and B4GalT1 as run on a native blue gel, while the rightmost lane shows a western blot analysis of the B4GalT1 sample detected with the B4GalT1 antibody. BSA fraction V (monomer size 66 kDa) and its oligomers (dimer, 133 kDa; trimer 200 kDa; tetramer 266 kDa) were used as a reference for molecular weight, as indicated in the figure in kDa.

The catalytic domain of B4GalT1 was expressed in *E*. *coli* and purified to homogeneity. The enzyme was active with an apparent *K*_M_ for the donor substrate UDP-Gal of 45.5 ± 5.4 μM, which falls within the range of previously published studies [19, 35–38], and an apparent *k*_cat_ of 0.059 ± 0.002 s^-1^. The oligomerization state of the recombinant B4GalT1 was investigated by two methods: gel filtration, and native blue gel followed by western blotting. The gel filtration profile revealed a major peak (Fig 1A, peak 2) and a minor peak (Fig 1A, peak 1), corresponding to the size of monomers and dimers, respectively. A sample taken from the monomer peak was analyzed on a native blue gel and it revealed both monomers and dimers (Fig 1B), indicating that B4GalT1 readily equilibrates between the two states.

### Structures of the wild-type B4GalT1

To date, several crystal structures of human B4GalT1 have been solved (S1 Table; [3, 14, 39, 40]). They all displayed an orthorhombic space group (C222_1_) and represented monomers, crystallographic dimers or trimers, and possessed mutations R337T, C338T and M340H. In addition, bovine B4GalT1 structures have been solved, containing also a wild-type enzyme structure (PDB code 1FR8) [12]. In the present work we used the wild-type human B4GalT1 and our construct is longer than the previously studied ones (Asp99-Ser398 instead of Ser126-Ser398). It contains a piece of the stem domain (Asp99-Leu125) in addition to the catalytic domain (Ser126-Ser398). We obtained two crystals with distinctly different shapes in the same crystallization conditions. These led to two different structures: (i) 50–200 μm sticks of orthorhombic crystal form (PDB code 6FWT), with one molecule per asymmetric unit, displaying an open conformation; (ii) 25–100 μm triangular prisms of trigonal crystal form (PDB code 6FWU), with two molecules per asymmetric unit, displaying a closed conformation. The refinement statistics are summarized in Table 1. Our construct contained residues 99–398, out of which residues 122–398 or 126–398 (in 6FWT and 6FWU, respectively) are observed in the electron density maps. This is consistent with residues 99–125 being part of the highly flexible and unstructured stem domain. However, an idea of the location of the missing N-termini of both open and closed conformation structures can be seen from the small-angle X-ray scattering envelope (S1 Fig).

**Fig 2:**
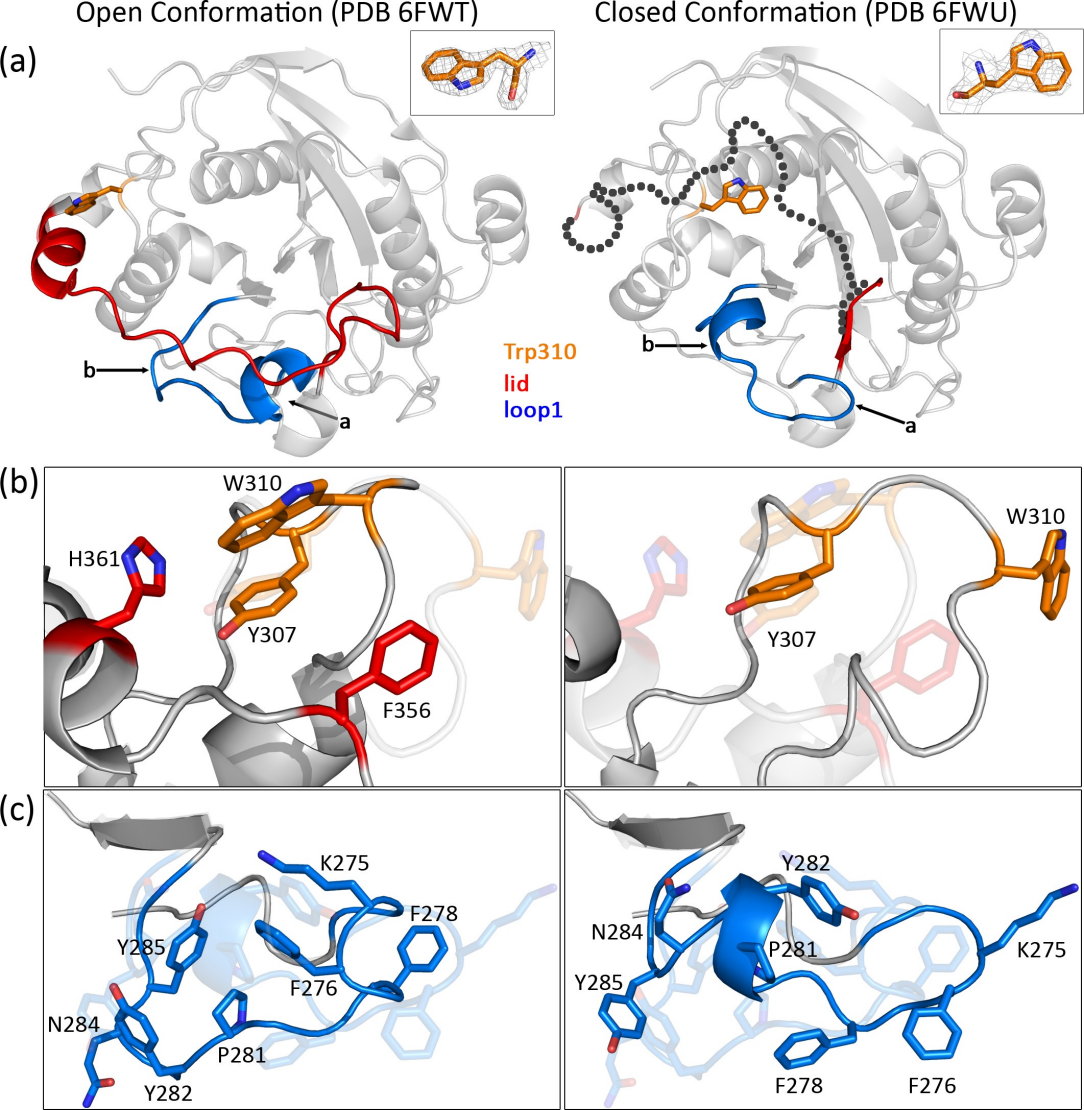
Structures of the human wild-type B4GalT1 in the open and closed conformation (left and right, PDB codes 6FWT and 6FWU, respectively). (a). Cartoon view of the structures. The highlighted areas are loop1 (blue), Trp loop (orange) and the lid (red). For loop1, the changes in secondary structure between open and closed structures are indicated with small arrows with “a” indicating residues 274–277 and “b” indicating residues 280–284, respectively. Trp310 is highlighted in orange sticks. Inset: Omit electron density map (2mFo-DFc) for Trp310. In the closed conformation structure the position of the closed lid (absent from the electron density), inferred by superimposition with the structure of the human mutated closed structure (PDB code 4EE4), is indicated as dashed black circles. (b, c). Close-up view of key amino acids in the vicinity of Trp310 and loop1, respectively. A faded image shows the corresponding structure in closed or open conformation or *vice versa* for easier comparison. One-letter code of amino acid residues is used for clarity due to space limitation in the figure.

In the monomeric open structure (PDB entry 6FWT), the electron density for the lid (amino acids 338–363) is very well defined and it was possible to reconstruct this entire lid region which was absent from the molecular replacement model (Fig 2A, red).

In the dimeric closed structure (PDB entry 6FWU), many of the lid residues have no clear electron density (amino acids 345–363) and could not be traced. Despite the absence of the lid in this structure, the position of the Trp loop (amino acids 308–311), with Trp310 facing the inside of the catalytic pocket, clearly indicates the presence of the closed conformation (Fig 2A and 2B). The RMSD value between the two monomers in the dimer is 0.210 Å.

Singular structural features never observed before can be described both in the open and closed conformation structures. The Trp loop in the open structure lies in the vicinity of His361 and Tyr307 and makes short distance (<3.8Å) hydrophobic contacts with those residues as well as long range (≈7Å) hydrophobic contacts with Phe356 (Fig 2B). In the case of the closed structure all of those contacts are replaced with a single short-range hydrophobic contact between Trp310 and Phe222. Another difference between the monomeric open and dimeric closed structures concerns loop1 (residues 272–288) (Fig 2A and 2C, blue). In the closed structure, we observe that loop1 is displaced deeper towards the catalytic pocket than in the open one, with an RMSD ≈ 6–10 Å (Fig 2C). Loop 1 not only exhibits a different position but also differences in secondary structure. Specifically, the one-turn α-helix usually formed by residues 274–277 in the open conformation becomes a β-turn in the closed conformation (Fig 2A, arrow a) and residues 280–284 transform from a random coil in the open conformation to a one-turn α-helix in the closed conformation (Fig 2A, arrow b). The observed position of loop1 in the closed structure is also such that the lid cannot be in the open conformation because of steric hindrance. This reinforces the conclusion that the observed structure represents the closed conformation of the lid, which is nevertheless flexible, as implied by the weak electron density.

### Dimeric structure of B4GalT1 in the closed conformation

**Fig 3:**
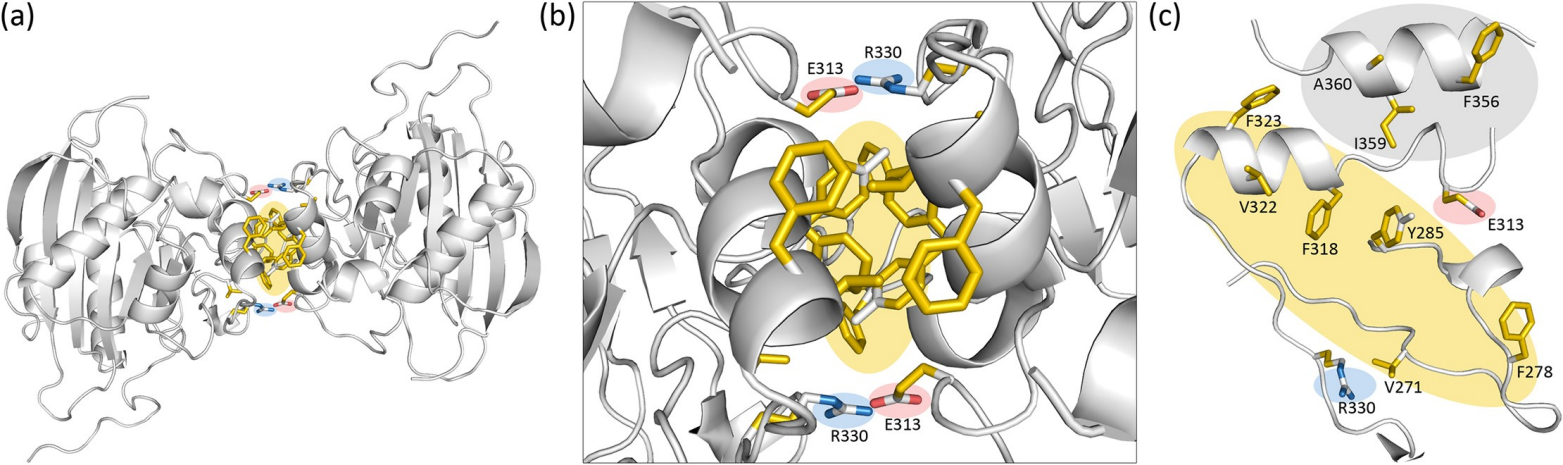
View of the dimer interface of human wild-type B4GalT1 as observed in the asymmetric unit of 6FWU (a): View of the dimer in a cartoon representation. The dimer interaction surface is highlighted in sticks in YRB coloring [54]: yellow (hydrophobic), red (negative), blue (positive), white (polar uncharged). (b, c): Detailed view of the interaction surface. Side chains of amino acids participating in the dimer interaction are shown in sticks and labeled. In panel (c), extra amino acids from the reconstructed lid (F356, I359, A360) involved in the interaction are depicted with gray background.

The closed structure shows two molecules per asymmetric unit (Fig 3A), with no significant conformation difference between the two chains. There is a symmetrical pair of salt bridges between Glu313 and Arg330, which lie at the rim of the dimer interface (Fig 3A and 3B). Between them reside hydrophobic contacts, which mostly maintain the dimerization interface: residues from loop1 (Val271, Gly277, Phe278 and Tyr285) and close to the Trp loop (Phe318, Val322 and Phe323) (Fig 3C). Based on the orientation of Trp310 and the position of loop1 the enzyme is in the closed conformation. We constructed a model of the homodimer including residues 345–363, not observed in the electron density, by merging the closed conformation lid (amino acids 341–365) of the mutated closed structure (PDB code 4EE4) into our wild-type closed structure (data not shown). This reconstruction did not generate clashes in the dimeric structure, but upon addition of the lid, three more hydrophobic contacts appeared between residues at the N-terminus of the lid (Phe356, Ile359, Ala360) and residues nearby the Trp loop (Gly325).

### Biological relevance of the B4GalT1 closed conformation dimer

The dimer observed in our closed conformation structure (6FWU) shows an interface area of 1106 Å^2^ when considering the reconstructed model containing the lid (Fig 3C) and has a total predicted binding energy value of -17.04 kcal/mol (Table 2, S3 Fig). These values compare well with values obtained for dimeric proteins considered biologically relevant with high confidence [29]. Similarly jsPISA and DiMoVo scores (Table 2) suggest a biologically relevant dimer. Note all of these are different when considering the structure without the reconstructed lid, but they less accurately describe the true situation. Multiple sequence alignments of the dimer interaction surface residues of human B4GalT1 and enzymes from other animals show a good conservation, either by identity or by homology, suggesting that the dimerization mechanism could be conserved between species (S4 Fig, S5 Fig and S7 Fig). This conservation is especially high in all GT family 7 enzymes for residues Glu313 and Arg330 which form the symmetrical salt bridges on opposite edges of the dimer interaction surface (S4 Fig, S5 Fig, S6 Fig and S7 Fig).

**Table 2:** Summary of the analysis of the dimer interface of the human wild-type B4GalT1 closed conformation crystal structure, which shows metrics in accordance with biological relevance. Metrics calculated by the jsPISA server are interface area (IA), solvation energy (DG) and total binding energy (BE). jsPISA score is a weighted average of each of the jsPISA radar metrics, for which a value higher than 50% depicts good chances of the interface being biologically relevant. DiMoVO score values below 0.5 predict crystallographic dimers, while values above 0.5 predict biological dimers.

PDB codes	Conformation	IA (Å^2^)	DG (kcal/mol)	BE (kcal/mol)	jsPISA	DiMoVo
**6FWU**	Closed	659.3	-10.11	-12.86	49%	0.3
**6FWU with the lid modelled**	Closed	1106	-12.96	-17.04	58%	0.5

### Identification of mutations abolishing the B4GalT1 homodimers *in vivo*

**Fig 4:**
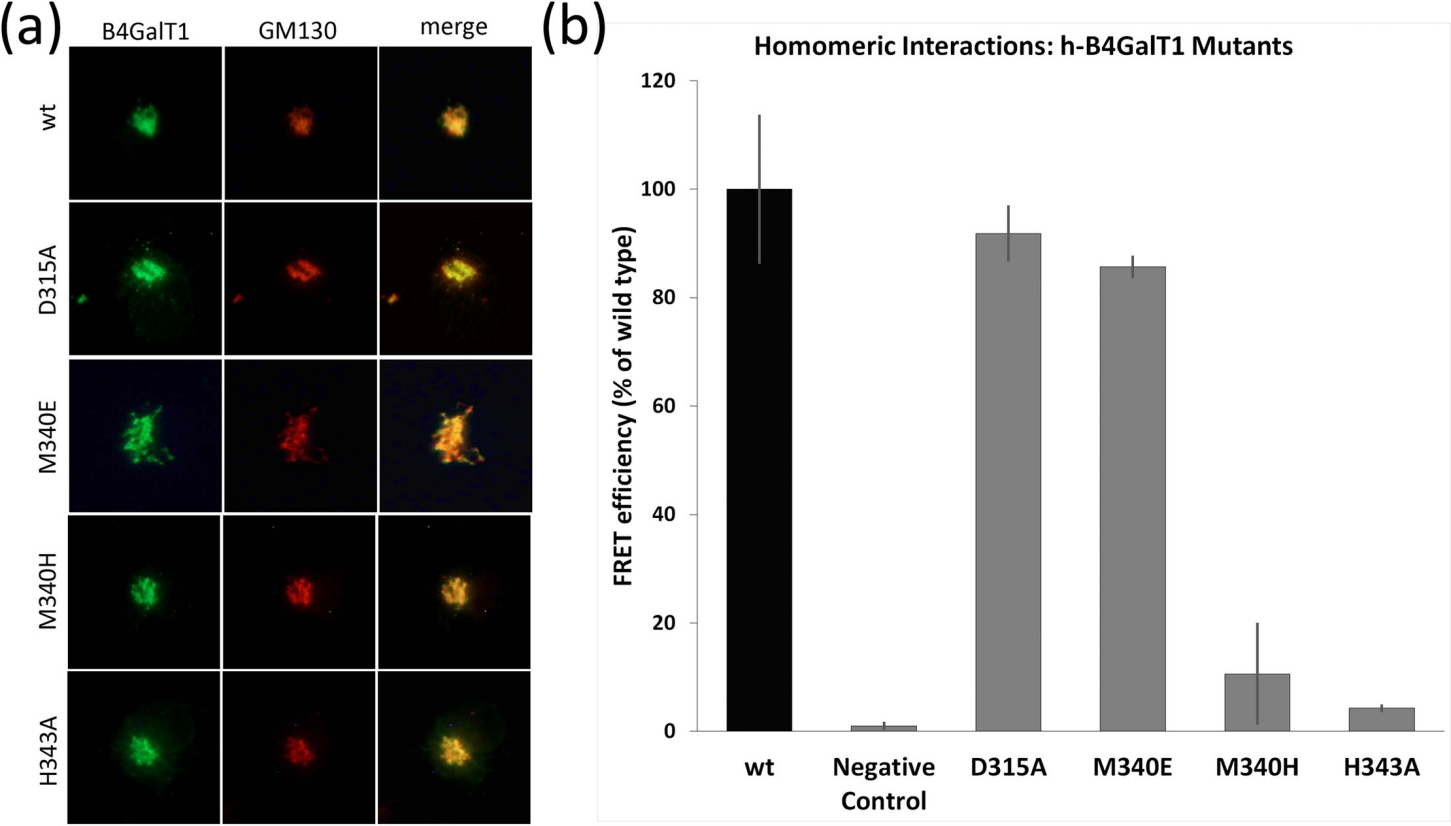
Dimer formation of full-length B4GalT1 type II membrane protein *in vivo*. (a). Localization of the human wild-type B4GalT1 and its variants in COS-7 cells. Green: B4GalT1-mVenus. Red: GM130 Golgi marker antibody as detected by Alexa Fluor 594 conjugated secondary antibody. Yellow: the previous two merged in the same figure. Scale bar, 10 μM. (b). FRET inhibition assays in COS-7 cells. Note the almost complete loss of homodimers with the M340H and H343A constructs. Error bars are calculated from triplicate experiments.

We next wanted to check whether B4GalT1’s activity and its ability to form homodimers were correlated. We selected key amino acids of the enzyme’s catalytic cycle to perform targeted mutagenesis studies coupled to FRET assays in COS-7 cells. Residue Asp315 was selected as a weak contactor of the acceptor sugar molecule. Residues Met340 and His343 were selected as weak and strong Mn^2+^ ion binding residues, respectively. The localization of the variant proteins was first verified. As all variants localized properly in the Golgi apparatus (Fig 4A) we concluded that the mutations did not impair normal folding. Transfection of the cells with mVenus and mCherry fused variants revealed that the single mutations D315A and M340E had no effect on dimer formation. In contrast, mutations M340H and H343A resulted in almost complete loss of the FRET signal (Fig 4B), indicating the loss of dimer formation.

Oligomerization was originally considered an important factor for Golgi localization and retention [41]. Here we show that variants M340H or H343A, which abolish B4GalT1 homomers formation, are still properly localized in the Golgi (Fig 4A).

## Discussion

### Structural differences in the lid, in the Trp loop and in loop1

**Fig 5:**
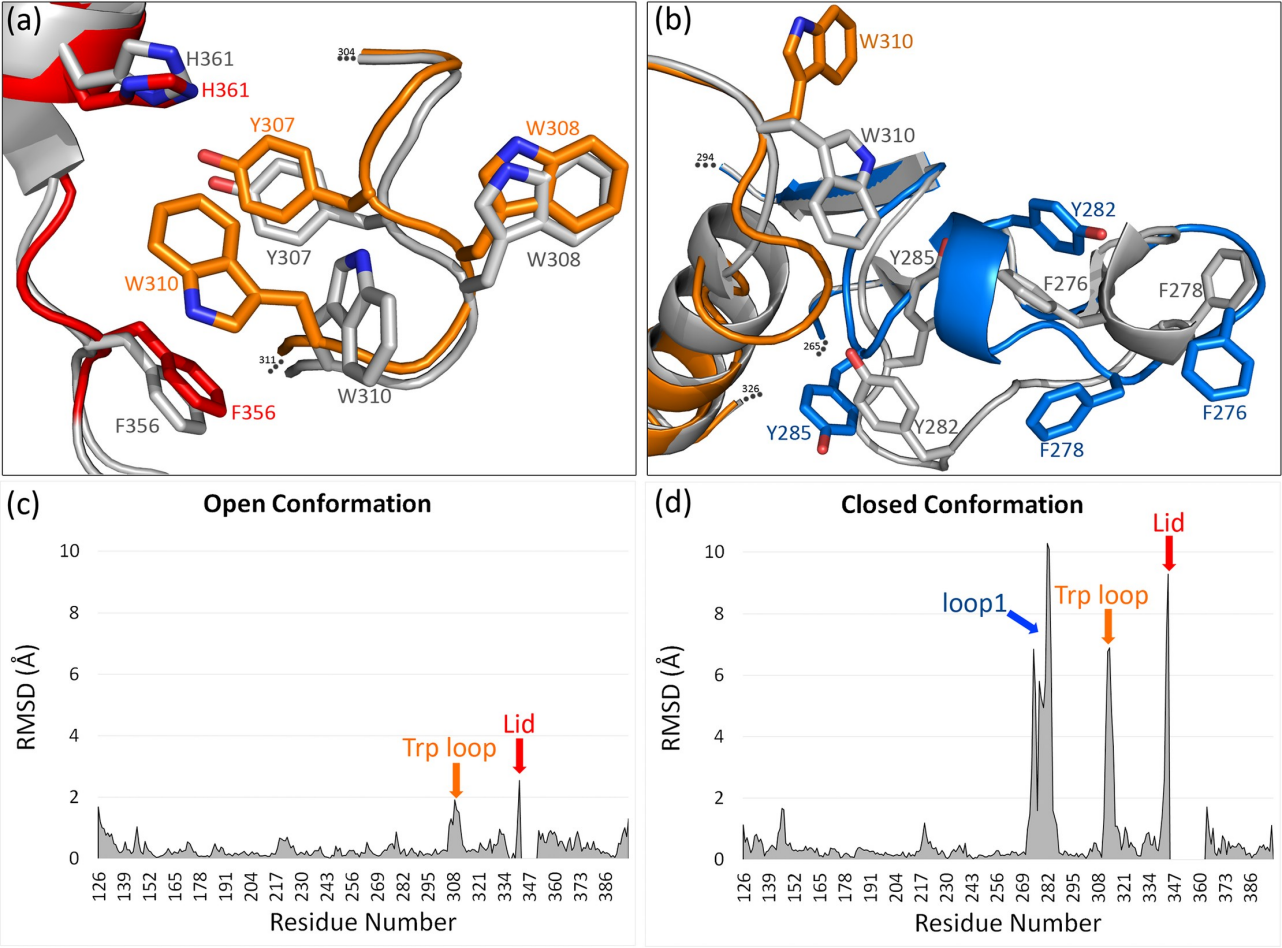
Structural differences between the wild-type and mutant B4GalT1 structures. (a). Details of the Trp loop and lid regions in the open wild-type structure (orange and red, respectively, PDB code 6FWT) and in the open mutated structure (gray, PDB code 2FY7). (b). Details of the Trp loop and loop1 regions in the closed wild-type structure (orange and blue, respectively, PDB code 6FWU) and closed mutated structure (gray, PDB code 4EE4). (c, d). Root mean square deviation (RMSD) of atomic positions in pairwise comparisons plotted by residue number between 6FWT and 2FY7 (c), and 6FWU and 4EE4 (d). Differences (RMSD > 2 Å) are highlighted by arrows. Residues that could not be traced in the crystal structures were omitted in the RMSD plot (343–351 in 2FY7, 345–363 in 6FWU).

In our open conformation structure of the human wild-type B4GalT1, the lid region, the most prominent ligand binding-controlling structural element, is detectable in its entirety. This was not the case in previously published structures of human open B4GalT1 [3], containing mutations R337T, C338T and M340H. However, comparison of the lid positions can be made with the bovine open B4GalT1 structure [12], for which all residues could be traced in the electron density map. Even though the proteins have crystallized in different space groups and unit cells and the packing of molecules is different, the lid is observed in a similar orientation, except for residues 346–352, which are slightly displaced (RMSD ≈ 2.5 Å, data not shown). One noteworthy difference which is functionally important for ligand binding, is that in our open structure, Trp310 has a new and unexpected orientation (Fig 5A) with an RMSD of ≈ 2.0 Å compared with human closed structure (Fig 5B).

There are strong arguments in favor of our dimer structure having the closed conformation. Firstly, loop1 is in a position such that it cannot coexist with the lid in the open conformation. Secondly, if loop1 was in the conformation seen in the structure of the mutated B4GalT1 [14] it would clash with the dimer, i.e. only one loop1 of the two monomers could be in the active conformation at the same time. There is also a possibility that the lid in our structure is in an intermediate conformation. Both scenarios include the possibility of loop1 from one monomer helping loop1 from the other monomer to undergo the conformational change from the open to the closed state. This proposed duality of loop1 could be part of concerted dynamics of all ligand-binding elements. It is known from the mutated closed structure that Phe276, Tyr282 and Tyr285 (residing in the loop1), Phe356 and Arg355 (residing in the lid), as well as Trp310, Asp314 and Asp315 (residing in the Trp loop) interact directly with the acceptor GlcNAc molecule. Furthermore, in our wild-type enzyme structure, part of the Trp loop (residues Gly312 and Glu313) and loop1 (Pro281, Val283 and Gln284) occupy the space normally used by an acceptor and residues Tyr282 and Tyr285 have altered orientations compared to those in the mutated closed structure. Of these, Tyr285 has been identified as a residue responsible for donor specificity [42]. Specifically, mutating this residue to a Phe does not change the activity, but mutation to a Gly diminishes the activity to below the detection limit [43]. The Trp loop is again in a very different (RMSD ≈ 6 Å; Fig 5C and 5D) and unexpected orientation compared to all the previously published structures. It is possible that the novel conformation of loop1 pushes Trp310 to a more remote position (Fig 5C).

Previously it had been assumed that the conformational change between the open and closed states could not happen without bound substrate [44]. Our structures demonstrate that it can and supports the hypothesis that transition from open to closed conformation is favored and can happen even with the unliganded enzyme.

### Concerted movements of Loop1, Trp loop and lid

The movements of the Trp loop and the lid during ligand-induced conformational changes have been shown to be highly coordinated [6, 10, 11, 13]. The communication of the flexibility of the Trp loop to the lid, through hydrophobic and hydrogen bonding interactions lowers the energy barrier for the transition between the opened and the closed conformation. When the Trp loop flips inside the binding pocket, the lid moves simultaneously over the donor substrate, masking the sugar nucleotide binding site and locking its position. This conformational change in the lid repositions Met340 and His343, creating the Mn^2+^ ion binding site and creating the 354–361 α-helix needed for the interaction with the acceptor oligosaccharide. Our current structure adds to this coordinated structural dynamics since it suggests that movements of the Trp loop and loop1 are also concerted. We propose that the lid needs hinge residues to trigger the movement between closed and open states, and residues Met340 and His361 are good candidates for this.

### Role of Mn^2+^ binding and the flexibility of the lid in homomerization

Key residues of the Mn^2+^ binding site, as found in the bovine enzyme, are Asp250 and His343 and Met340, which coordinates less strongly [9, 45]. We have shown here that H343A abolishes the formation of B4GalT1 homomers *in vivo*. We interpret this so that removing one of the two strong ligands leaves inadequate ability for Mn^2+^ to stay in place. We also show that M340H, but not M340E, reduces drastically the stability of B4GalT1 homomers. The M340H variant of the bovine enzyme has been shown to be 25 times more efficient in Mn^2+^ binding, but possesses only 2% of the wild-type enzyme activity [9]. This is straightforward to understand as the M340H mutation blocks B4GalT1 in the closed conformation and thus seriously affects the dynamics of the lid. This appears also to have been the fortuitous reason for crystallizability of B4GalT1 in previous studies. In contrast, the M340E variant does not lock the Mn^2+^ ion to the enzyme and although such a mutation reduces activity by 94% [9] the dynamics of the lid are preserved, and so is homomerization.

### Functional relevance of B4GalT1 homodimers

**Fig 6:**
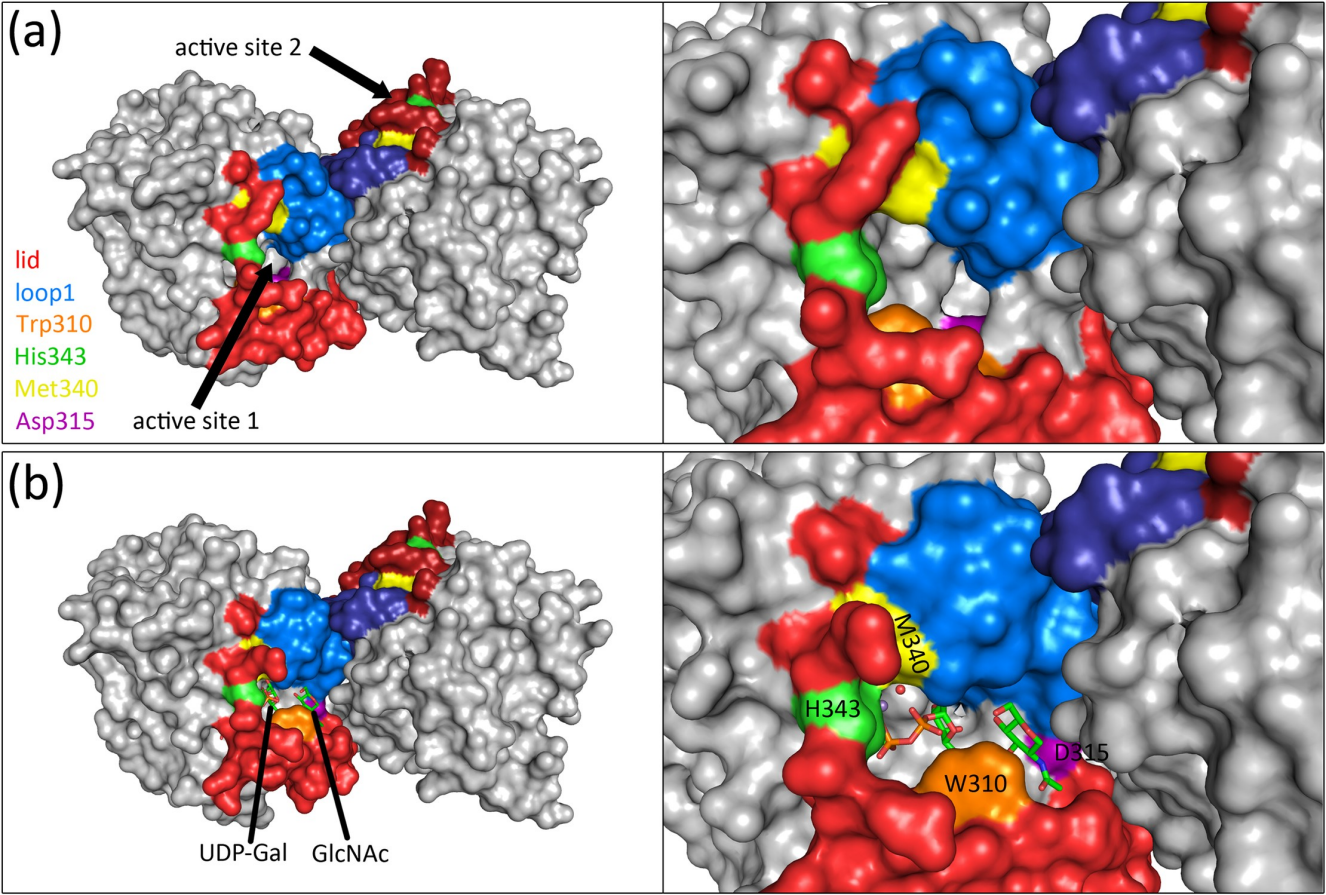
Functional relevance of the B4GalT1 homodimers. Monomers within the dimer are displayed with the surface representation and colored gray. Key residues and regions are highlighted as follows: Lid (red), loop1 (blue), Trp310 (orange), Asp315 (purple), Met340 (yellow), His343 (green). Darker color tints are used for the second monomer in the dimer. (a). Relative positions of the active site positions (black arrows) in the B4GalT1 homodimer structure (PDB code 6FWU). (b). Model of a B4GalT1 dimer with one chain in an active conformation (including the donor UDP-Gal, acceptor GlcNAc and Mn^2+^ ion from PDB codes 1O0R and 4EE4, respectively) on the B4GalT1 homodimer structure (PDB code 6FWU) with reconstructed lid.

In the closed dimer of the wild-type B4GalT1, the H343 active site residues are 35.5 Å apart. The active sites are slightly tilted relative to each other and there is sufficient space for reaction to occur (Fig 6A).

In the presence of substrates and in the closed conformation, the Mn^2+^ ion is observed bound at the N-terminal part of the lid. It shows hexa-coordination by residues Met340 and His343 from the lid, Asp248 and Asp250 ([46]; the bond with Asp248 being mediated through a water molecule), supplemented by two interactions with the UDP-Gal molecule, one to each of the phosphate group oxygen [6, 14]. The Gal moiety of UDP-Gal is often hydrolyzed during crystallization, but has been observed coordinated by Glu313, Arg224, Asp248 and Gly288 [3, 9, 47, 48]. The UDP moiety is coordinated with the Mn^2+^ ion, Trp310, Arg187 and Asp250, and by the small hydrophobic pocket formed by Pro183, Phe184, Phe222, Val249 and Leu251 [14]. The acceptor GlcNAc molecule is maintained by hydrophobic interactions with Phe276 and Tyr282 from loop1, which are partially buried in the open conformation but accessible in the closed conformation [9, 47]. Its N-acetyl group is bound to residues Arg355, Phe356 and Ile359, which are part of the closed-form α-helix at the C-terminal end of the lid.

To aggregate these data we modelled a B4GalT1 dimer with its substrates, by merging one chain in active substrate-bound conformation (including the acceptor GlcNAc, the donor UDP-Gal and the Mn^2+^ ion) onto our closed dimer (Fig 6B). Interestingly, this model places the acceptor GlcNAc ≈6Å from the dimerization interface, such that the second monomer of the dimer could help binding it through mid-range contacts. In this model these could likely come from residues 328–334.

It is well established that GTases including B4GalT1 form homomers in the ER [22] mainly via their catalytic domains [49, 50], and perhaps also via parts of the stem domains [51–53]. They also undergo a transition to form enzyme heteromers with a relevant enzyme partner upon arrival of the homomers in the Golgi e.g. in the case of B4GalT1 with ST6Gal1. This transition is likely driven by the different internal milieu, for example pH, of the Golgi lumen relative to that of the ER [22]. The homodimer structure solved here most closely mimics the one that normally exists in the ER, or alternatively, in the Golgi in cells overexpressing only one of the players needed for heteromer formation. Therefore, the B4GalT1 dimer structure represents an active, yet short term, enzyme species in mammalian cells, but that can also persist in the Golgi in the absence of an adequate amount of ST6Gal1. An important question is therefore to find out whether ST6Gal1 uses the same or a different binding interface in B4GalT1 than the B4GalT1 uses for homodimerization. Site-directed mutations and variant stem domains could be instrumental in clarifying molecular events in glycosyltransferase dimerization further.

## Supporting information

S1 Table Previously published B4GalT1 structures. (DOCX)

S1 Text FRET plasmid constructs and their preparation. (DOCX)

S2 Text Full wwPDB X-ray structure validation report for 6FWT. (PDF)

S3 Text Full wwPDB X-ray structure validation report for 6FWU. (PDF)

S1 Fig Gene sequence of B4GalT1 used for cloning and expression in E. coli. (DOCX)

S2 Fig Small-angle X-ray scattering (SAXS) envelope to locate N-termini not seen in the crystal structures. (DOCX)

S3 Fig jsPISA analysis of homodimer interface of B4GalT1 (PDB code 6FWU). (DOCX)

S4 Fig Conservation of the residues at the dimerization interface observed in the closed structure of B4GalT1 (PDB code 6FWU). (DOCX)

S5 Fig Multiple sequence alignment of the human members of the GalT family. (DOCX)

S6 Fig Multiple sequence alignment of the human members of the GT7 family. (DOCX)

S7 Fig Multiple sequence alignment of the B4GalT1 protein among animal species. (DOCX)

## Abbreviations

BSA: bovine serum albumin
FRET: fluorescence resonance energy transfer
Gal: galactose
GlcNAc: N-acetylgalactosamine
PBS: phosphate buffered saline
PDB: protein data bank
RMSD: root mean square deviation
UDP: uridine diphosphate
UDP-Gal: uridine diphosphate galactose
