# Supplementary material for: The dimeric structure of wild-type human glycosyltransferase B4GalT1
Source: PLoS One. 2018 Oct 23;13(10):e0205571. doi: 10.1371/journal.pone.0205571 (PMC6198961; doi:10.1371/journal.pone.0205571)
Supplement: S1 Table — (DOCX) [file pone.0205571.s001.docx]

**S1 Table. Previously published B4GalT1 structures**

| **Organism** | **PDB codes** | **Conformation** | **Mutations** | **Chains** | **IA (Å²)** | **DG (kcal/mol)** | **BE (kcal/mol)** | **jsPISA** | **DiMoVo** | **Reference** |
| --- | --- | --- | --- | --- | --- | --- | --- | --- | --- | --- |
| **Human** | **2FY7**, 2FYA, 2FYB | Open | R337T, C338T, M340H | **A**(**A'**) | 612.7 | -10.06 | -11.83 | 49 | 0.4 | Ramakrishnan 2006 JMB |
|  | 2AE7, 2AEC, 2AES, **2AGD**, 2AH9 | Closed | R337T, C338T, M340H | **A**/B/**C** | 471.5 | -8.005 | -8.005 | 34 | 0.0 | Ramasamy 2005 JMB |
|  | 3EE5, 4EE3, **4EE4**, 4EE5, E44A, 4EEG, 4EEM, 4EEO | Closed | R337T, C338T, M340H | **A**/B/**C** | 458.4 | -8.385 | -8.385 | 36 | 0.0 | Brown 2009 JBC,  Ramakrishnan 2012 JBC |
| **Bovine** | **1FGX**, 1FR8 | Open | V158I / none | **A**/**B** | 742,6 | -6.142 | -11.63 | 46 | 0.3 | Gastinel 1999 EMBO |
|  | **1O0R** | Closed | C342T | **A**/**B** | 498.8 | -5.694 | -8.359 | 31 | 0.0 | Ramakrishnan 2002 JMB |
|  | **1PZT** | Intermediate | W314A, C342T | **A**(**A'**) | 679.6 | -8.38 | -10.16 | 40 | 0.2 | Ramasamy 2003 JMB |
|  | **1TVY**, 1TW1, 1TW5 | Closed | C342T, M344H | **A**/**B** | 502.2 | -4.668 | -7.776 | 29 | 0.1 | Ramakrishnan 2004 Biochemistry |
|  | **4KRV** | Closed | C342T, M344H | **A**/**B** | 491.6 | -5.208 | -7.428 | 27 | 0.1 | Ramakrishnan 2013 Glycoconj |

Summary of the analysis of the dimer interface of the previously published crystal structures of B4GalT1. A large number of structures showed an absence (jsPISA score < 50%, DiMoVO score < 0.4) of biologically relevant protein-protein contacts within the asymmetric unit, or with symmetry molecules. Structures are grouped by cell packing type; the structure with the best resolution in each group (PDB code in bold) was analyzed for potential biologically relevant dimers. Bolded chains indicates the name of the chains in the crystal structure used to analyse the dimer interface. Monomers related by a crystallographic symmetry are written in brackets in opposition to monomers related by a non-crystallographic symmetry. When more than two chains were present in the asymmetric unit (i.e. A, B and C), all chain pairs were tested (AB, AC, BC) and the pair giving the highest jsPISA score was selected. IA, interface area; DG, solvation energy; BE, total binding energy. jsPISA score is a weighted average of each of the jsPISA radar metrics, for which a value higher than 50% depicts good chances of the interface being biologically relevant. DiMoVO score values below 0.5 predict crystal dimers, while values above 0.5 predict biological dimers.
