# Supplementary material for: The dimeric structure of wild-type human glycosyltransferase B4GalT1
Source: PLoS One. 2018 Oct 23;13(10):e0205571. doi: 10.1371/journal.pone.0205571 (PMC6198961; doi:10.1371/journal.pone.0205571)
Supplement: S1 Text — (DOCX) [file pone.0205571.s002.docx]

**S1 Text. FRET plasmid constructs and their preparation.**

The wild-type FRET plasmid constructs (B4GalT1-mVenus and – mCherry) were prepared as described previously (ref. 20 of the article). The variant B4GalT1-mVenus and –mCherry FRET plasmids (B4GalT1-D315A, B4GalT1-M340H, B4GalT1-M340E and B4GalT1-H343A) were generated by mutating key amino acid residues in the dimer interface by using site directed mutagenesis kit (QuickChange Lightning Site-Directed Mutagenesis Kit, Agilent). In brief, primers (see below) containing one or two base pair mutations were used for the PCR and sub cloned into the pcDNA3 plasmid containing either mVenus (YFP) tag or mCherry (RFP) tag using the HindIII (5’ cloning site) and XbaI (3’ cloning site) restriction enzymes. The mutant plasmids were transformed to XL10 gold *E. coli* strain before selecting the clones in ampicillin plates.

| Mutant | Primers |
| --- | --- |
| D315A Frw | GGCTGGGGAGGAGAAGATGCTGACATTTTTAACAGATTAG |
| D315A Rev | CTAATCTGTTAAAAATGTCAGCATCTTCTCCTCCCCAGCC |
| M340H Frw | GTCGGGAGGTGTCGCCACATCCGCCACTCAAGAGA |
| M340H Rev | TCTCTTGAGTGGCGGATGTGGCGACACCTCCCGAC |
| M340E Frw | GGTCGGGAGGTGTCGCGAGATCCGCCACTCAAGAGAC |
| M340E Rev | GTCTCTTGAGTGGCGGATCTCGCGACACCTCCCGACC |
| H343A Frw | GGAGGTGTCGCATGATCCGCGCCTCAAGAGACAAGAAAAATG |
| H343A Rev | CATTTTTCTTGTCTCTTGAGGCGCGGATCATGCGACACCTCC |
