# Supplementary material for: The dimeric structure of wild-type human glycosyltransferase B4GalT1
Source: PLoS One. 2018 Oct 23;13(10):e0205571. doi: 10.1371/journal.pone.0205571 (PMC6198961; doi:10.1371/journal.pone.0205571)
Supplement: S3 Text — (PDF) [file pone.0205571.s004.pdf]

Full wwPDB X-ray Structure Validation Report (i)

Mar 7, 2018 – 03:06 PM GMT

PDB ID	:	6FWU
Title	:	Crystal structure of human wild type beta-1,4-galactosyltransferase-1 (B4GalT1) in apo-closed dimeric form
Deposited on	:	2018-03-07
Resolution	:	2.35 Å(reported)

This is a Full wwPDB X-ray Structure Validation Report.

This report is produced by the wwPDB biocuration pipeline after annotation of the structure.

We welcome your comments at *validation@mail.wwpdb.org*
A user guide is available at
http://wwpdb.org/validation/2016/XrayValidationReportHelp
with specific help available everywhere you see the (i) symbol.

CONFIDENTIAL VALIDATION REPORT

The following versions of software and data (see references (i)) were used in the production of this report:

MolProbity	:	4.02b-467
Mogul	:	1.7.3 (157068), CSD as539be (2018)
Xtriage (Phenix)	:	1.9-1692
EDS	:	rb-20030736
Percentile statistics	:	20161228.v01 (using entries in the PDB archive December 28th 2016)
Refmac	:	5.8.0135
CCP4	:	6.5.0
Ideal geometry (proteins)	:	Engh & Huber (2001)
Ideal geometry (DNA, RNA)	:	Parkinson et al. (1996)
Validation Pipeline (wwPDB-VP)	:	rb-20030736

1 Overall quality at a glance (i)

The following experimental techniques were used to determine the structure:
X-RAY DIFFRACTION

The reported resolution of this entry is 2.35 Å.

Percentile scores (ranging between 0-100) for global validation metrics of the entry are shown in the following graphic. The table shows the number of entries on which the scores are based.

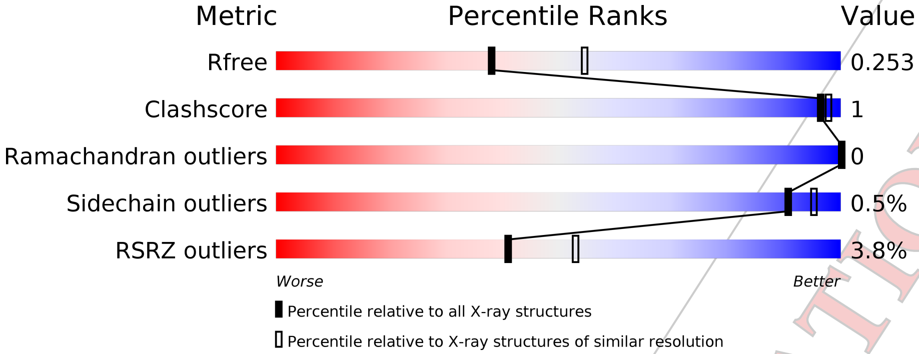

Metric	Whole archive (#Entries)	Similar resolution (#Entries, resolution range(Å))
R_{free}	100719	1522 (2.38-2.34)
Clashscore	112137	1626 (2.38-2.34)
Ramachandran outliers	110173	1605 (2.38-2.34)
Sidechain outliers	110143	1606 (2.38-2.34)
RSRZ outliers	101464	1528 (2.38-2.34)

The table below summarises the geometric issues observed across the polymeric chains and their fit to the electron density. The red, orange, yellow and green segments on the lower bar indicate the fraction of residues that contain outliers for >=3, 2, 1 and 0 types of geometric quality criteria. A grey segment represents the fraction of residues that are not modelled. The numeric value for each fraction is indicated below the corresponding segment, with a dot representing fractions <=5% The upper red bar (where present) indicates the fraction of residues that have poor fit to the electron density. The numeric value is given above the bar.

Mol	Chain	Length	Quality of chain
1	A	273	4% / 89% • 7%
1	B	273	3% / 89% • 8%

The following table lists non-polymeric compounds, carbohydrate monomers and non-standard residues in protein, DNA, RNA chains that are outliers for geometric or electron-density-fit criteria:

CONFIDENTIAL VALIDATION REPORT

Mol	Type	Chain	Res	Chirality	Geometry	Clashes	Electron density
2	NO3	A	401	-	-	-	X

CONFIDENTIAL VALIDATION REPORT

2 Entry composition (i)

There are 3 unique types of molecules in this entry. The entry contains 7859 atoms, of which 3803 are hydrogens and 0 are deuteriums.

In the tables below, the ZeroOcc column contains the number of atoms modelled with zero occupancy, the AltConf column contains the number of residues with at least one atom in alternate conformation and the Trace column contains the number of residues modelled with at most 2 atoms.

- Molecule 1 is a protein called Beta-1,4-galactosyltransferase 1.

Mol	Chain	Residues	Atoms						ZeroOcc	AltConf	Trace
			Total	C	H	N	O	S			
1	A	253	3917	1293	1906	346	359	13	0	0	0
1	B	251	3884	1280	1897	338	356	13	0	0	0

- Molecule 2 is NITRATE ION (three-letter code: NO3) (formula: NO_3).

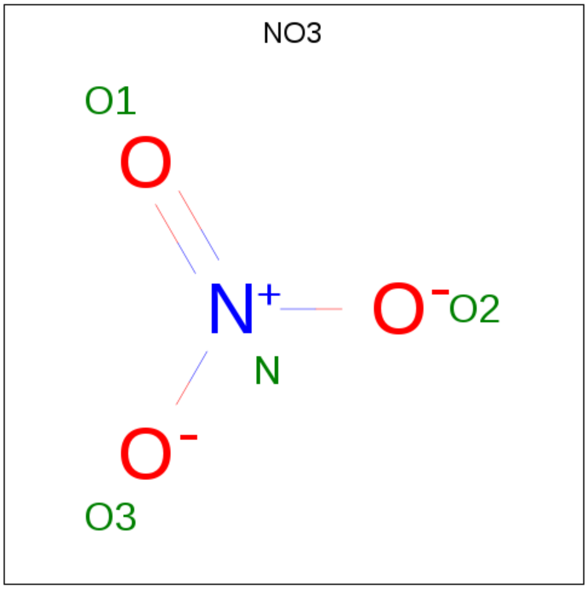

Mol	Chain	Residues	Atoms			ZeroOcc	AltConf
			Total	N	O		
2	A	1	4	1	3	0	0
2	B	1	4	1	3	0	0

- Molecule 3 is water.

CONFIDENTIAL VALIDATION REPORT

Mol	Chain	Residues	Atoms		ZeroOcc	AltConf
3	A	21	Total<br>21	O<br>21	0	0
3	B	28	Total<br>29	O<br>29	0	1

CONFIDENTIAL VALIDATION REPORT

3 Residue-property plots ⓘ

These plots are drawn for all protein, RNA and DNA chains in the entry. The first graphic for a chain summarises the proportions of the various outlier classes displayed in the second graphic. The second graphic shows the sequence view annotated by issues in geometry and electron density. Residues are color-coded according to the number of geometric quality criteria for which they contain at least one outlier: green = 0, yellow = 1, orange = 2 and red = 3 or more. A red dot above a residue indicates a poor fit to the electron density (RSRZ > 2). Stretches of 2 or more consecutive residues without any outlier are shown as a green connector. Residues present in the sample, but not in the model, are shown in grey.

- Molecule 1: Beta-1,4-galactosyltransferase 1

Chain A:

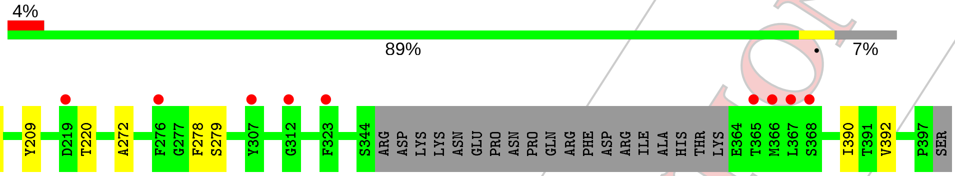

- Molecule 1: Beta-1,4-galactosyltransferase 1

Chain B:

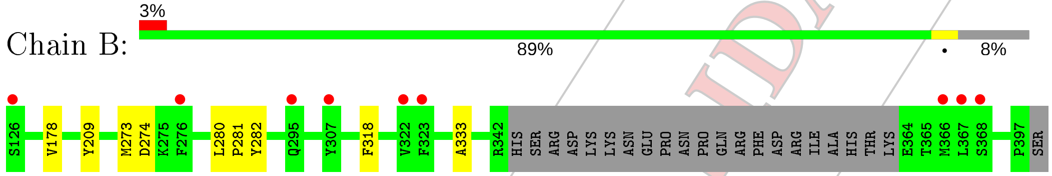

CONFIDENTIAL VALIDATION REPORT

4 Data and refinement statistics (i)

Property	Value	Source
Space group	P 31 2 1	Depositor
Cell constants<br>a, b, c, α, β, γ	60.22Å 60.22Å 229.37Å<br>90.00° 90.00° 120.00°	Depositor
Resolution (Å)	47.47 – 2.35<br>47.47 – 2.35	Depositor<br>EDS
% Data completeness<br>(in resolution range)	99.8 (47.47-2.35)<br>99.9 (47.47-2.35)	Depositor<br>EDS
R_{merge}	(Not available)	Depositor
R_{sym}	(Not available)	Depositor
$< I/\sigma(I) >$ [1]	1.59 (at 2.34Å)	Xtriage
Refinement program	PHENIX 1.11.1_2575	Depositor
R, R_{free}	0.195 , 0.256<br>0.193 , 0.253	Depositor<br>DCC
R_{free} test set	1053 reflections (5.01%)	DCC
Wilson B-factor (Å^2)	35.0	Xtriage
Anisotropy	0.446	Xtriage
Bulk solvent k_{sol}(e/Å^3), B_{sol}(Å^2)	0.40 , 38.4	EDS
L-test for twinning[2]	$< \|L\| > = 0.50$, $< L^2 > = 0.33$	Xtriage
Estimated twinning fraction	0.036 for -h,-k,l	Xtriage
F_o,F_c correlation	0.94	EDS
Total number of atoms	7859	wwPDB-VP
Average B, all atoms (Å^2)	41.0	wwPDB-VP

Xtriage's analysis on translational NCS is as follows: *The largest off-origin peak in the Patterson function is 5.62% of the height of the origin peak. No significant pseudotranslation is detected.*

[1] Intensities estimated from amplitudes.

[2] Theoretical values of $< |L| >$, $< L^2 >$ for acentric reflections are 0.5, 0.333 respectively for untwinned datasets, and 0.375, 0.2 for perfectly twinned datasets.

CONFIDENTIAL VALIDATION REPORT

5 Model quality (i)

5.1 Standard geometry (i)

Bond lengths and bond angles in the following residue types are not validated in this section: NO3

The Z score for a bond length (or angle) is the number of standard deviations the observed value is removed from the expected value. A bond length (or angle) with $|Z| > 5$ is considered an outlier worth inspection. RMSZ is the root-mean-square of all Z scores of the bond lengths (or angles).

Mol	Chain	Bond lengths		Bond angles	
		RMSZ	$\#|Z|>5$	RMSZ	$\#|Z|>5$
1	A	0.25	0/2066	0.46	0/2811
1	B	0.25	0/2041	0.46	0/2778
All	All	0.25	0/4107	0.46	0/5589

There are no bond length outliers.

There are no bond angle outliers.

There are no chirality outliers.

There are no planarity outliers.

5.2 Too-close contacts (i)

In the following table, the Non-H and H(model) columns list the number of non-hydrogen atoms and hydrogen atoms in the chain respectively. The H(added) column lists the number of hydrogen atoms added and optimized by MolProbity. The Clashes column lists the number of clashes within the asymmetric unit, whereas Symm-Clashes lists symmetry related clashes.

Mol	Chain	Non-H	H(model)	H(added)	Clashes	Symm-Clashes
1	A	2011	1906	1932	6	0
1	B	1987	1897	1908	4	0
2	A	4	0	0	0	0
2	B	4	0	0	0	0
3	A	21	0	0	0	0
3	B	29	0	0	0	0
All	All	4056	3803	3840	9	0

The all-atom clashscore is defined as the number of clashes found per 1000 atoms (including hydrogen atoms). The all-atom clashscore for this structure is 1.

All (9) close contacts within the same asymmetric unit are listed below, sorted by their clash

CONFIDENTIAL VALIDATION REPORT

magnitude.

Atom-1	Atom-2	Interatomic distance (Å)	Clash overlap (Å)
1:A:186:ASN:O	1:A:187:ARG:NH1	2.02	0.93
1:B:273:MET:HG3	1:B:282:TYR:CD2	2.45	0.52
1:A:178:VAL:O	1:A:209:TYR:HA	2.17	0.45
1:A:185:ARG:NH1	1:A:220:THR:O	2.47	0.44
1:A:390:ILE:HG22	1:A:392:VAL:HG13	2.00	0.43
1:A:278:PHE:CE2	1:B:333:ALA:HB2	2.53	0.43
1:B:178:VAL:O	1:B:209:TYR:HA	2.19	0.41
1:A:272:ALA:HA	1:A:279:SER:OG	2.21	0.41
1:B:280:LEU:N	1:B:281:PRO:CD	2.84	0.40

There are no symmetry-related clashes.

5.3 Torsion angles (i)

5.3.1 Protein backbone (i)

In the following table, the Percentiles column shows the percent Ramachandran outliers of the chain as a percentile score with respect to all X-ray entries followed by that with respect to entries of similar resolution.

The Analysed column shows the number of residues for which the backbone conformation was analysed, and the total number of residues.

Mol	Chain	Analysed	Favoured	Allowed	Outliers	Percentiles	
1	A	249/273 (91%)	239 (96%)	10 (4%)	0	100	100
1	B	247/273 (90%)	242 (98%)	5 (2%)	0	100	100
All	All	496/546 (91%)	481 (97%)	15 (3%)	0	100	100

There are no Ramachandran outliers to report.

5.3.2 Protein sidechains (i)

In the following table, the Percentiles column shows the percent sidechain outliers of the chain as a percentile score with respect to all X-ray entries followed by that with respect to entries of similar resolution.

The Analysed column shows the number of residues for which the sidechain conformation was analysed, and the total number of residues.

CONFIDENTIAL VALIDATION REPORT

Mol	Chain	Analysed	Rotameric	Outliers	Percentiles	
1	A	215/244 (88%)	215 (100%)	0	100	100
1	B	212/244 (87%)	210 (99%)	2 (1%)	82	90
All	All	427/488 (88%)	425 (100%)	2 (0%)	91	95

All (2) residues with a non-rotameric sidechain are listed below:

Mol	Chain	Res	Type
1	B	274	ASP
1	B	318	PHE

Some sidechains can be flipped to improve hydrogen bonding and reduce clashes. There are no such sidechains identified.

5.3.3 RNA (i)

There are no RNA molecules in this entry.

5.4 Non-standard residues in protein, DNA, RNA chains (i)

There are no non-standard protein/DNA/RNA residues in this entry.

5.5 Carbohydrates (i)

There are no carbohydrates in this entry.

5.6 Ligand geometry (i)

2 ligands are modelled in this entry.

In the following table, the Counts columns list the number of bonds (or angles) for which Mogul statistics could be retrieved, the number of bonds (or angles) that are observed in the model and the number of bonds (or angles) that are defined in the Chemical Component Dictionary. The Link column lists molecule types, if any, to which the group is linked. The Z score for a bond length (or angle) is the number of standard deviations the observed value is removed from the expected value. A bond length (or angle) with $|Z| > 2$ is considered an outlier worth inspection. RMSZ is the root-mean-square of all Z scores of the bond lengths (or angles).

Mol	Type	Chain	Res	Link	Bond lengths			Bond angles		
					Counts	RMSZ	$\#\|Z\| > 2$	Counts	RMSZ	$\#\|Z\| > 2$
2	NO3	A	401	-	1,3,3	0.58	0	0,3,3	0.00	-
2	NO3	B	401	-	1,3,3	0.59	0	0,3,3	0.00	-

CONFIDENTIAL VALIDATION REPORT

In the following table, the Chirals column lists the number of chiral outliers, the number of chiral centers analysed, the number of these observed in the model and the number defined in the Chemical Component Dictionary. Similar counts are reported in the Torsion and Rings columns. '-' means no outliers of that kind were identified.

Mol	Type	Chain	Res	Link	Chirals	Torsions	Rings
2	NO3	A	401	-	-	0/0/0/0	0/0/0/0
2	NO3	B	401	-	-	0/0/0/0	0/0/0/0

There are no bond length outliers.

There are no bond angle outliers.

There are no chirality outliers.

There are no torsion outliers.

There are no ring outliers.

No monomer is involved in short contacts.

5.7 Other polymers (i)

There are no such residues in this entry.

5.8 Polymer linkage issues (i)

There are no chain breaks in this entry.

CONFIDENTIAL VALIDATION REPORT

6 Fit of model and data (i)

6.1 Protein, DNA and RNA chains (i)

In the following table, the column labelled '#RSRZ> 2' contains the number (and percentage) of RSRZ outliers, followed by percent RSRZ outliers for the chain as percentile scores relative to all X-ray entries and entries of similar resolution. The OWAB column contains the minimum, median, 95th percentile and maximum values of the occupancy-weighted average B-factor per residue. The column labelled 'Q< 0.9' lists the number of (and percentage) of residues with an average occupancy less than 0.9.

Mol	Chain	Analysed	<RSRZ>	#RSRZ>2			OWAB(Å^2)	Q<0.9
1	A	253/273 (92%)	0.20	10 (3%)	39	51	23, 36, 62, 89	0
1	B	251/273 (91%)	0.09	9 (3%)	43	55	23, 35, 64, 134	0
All	All	504/546 (92%)	0.15	19 (3%)	41	53	23, 35, 63, 134	0

All (19) RSRZ outliers are listed below:

Mol	Chain	Res	Type	RSRZ
1	B	367	LEU	5.5
1	B	323	PHE	4.1
1	A	307	TYR	3.8
1	B	368	SER	3.7
1	B	295	GLN	3.7
1	A	276	PHE	3.6
1	A	365	THR	3.5
1	A	323	PHE	3.2
1	A	367	LEU	3.2
1	B	126	SER	3.1
1	A	368	SER	2.8
1	A	312	GLY	2.8
1	B	307	TYR	2.7
1	A	366	MET	2.7
1	A	126	SER	2.4
1	A	219	ASP	2.3
1	B	366	MET	2.2
1	B	322	VAL	2.2
1	B	276	PHE	2.0

6.2 Non-standard residues in protein, DNA, RNA chains (i)

There are no non-standard protein/DNA/RNA residues in this entry.

CONFIDENTIAL VALIDATION REPORT

6.3 Carbohydrates (i)

There are no carbohydrates in this entry.

6.4 Ligands (i)

In the following table, the Atoms column lists the number of modelled atoms in the group and the number defined in the chemical component dictionary. LLDF column lists the quality of electron density of the group with respect to its neighbouring residues in protein, DNA or RNA chains. The B-factors column lists the minimum, median, 95th percentile and maximum values of B factors of atoms in the group. The column labelled 'Q< 0.9' lists the number of atoms with occupancy less than 0.9.

Mol	Type	Chain	Res	Atoms	RSCC	RSR	LLDF	B-factors($Å^2$)	Q<0.9
2	NO3	A	401	4/4	0.94	0.20	2.73	45,45,48,53	0
2	NO3	B	401	4/4	0.98	0.16	0.76	39,45,46,46	0

6.5 Other polymers (i)

There are no such residues in this entry.

CONFIDENTIAL VALIDATION REPORT
