# Supplementary material for: The dimeric structure of wild-type human glycosyltransferase B4GalT1
Source: PLoS One. 2018 Oct 23;13(10):e0205571. doi: 10.1371/journal.pone.0205571 (PMC6198961; doi:10.1371/journal.pone.0205571)
Supplement: S1 Fig — (DOCX) [file pone.0205571.s005.docx]

**S1 Fig. Gene sequence of B4GalT1 used for cloning and expression in E. coli**

**CATATG**GACTCCAGCCCAGTCGTGGATTCTGGCCCTGGCCCCGCTAGCAACTTGACCTCGGTCCCAGTGCCCCACACCACCGCACTGTCGCTGCCCGCCTGCCCTGAGGAGTCCCCGCTGCTTGTGGGCCCCATGCTGATTGAGTTTAACATGCCTGTGGACCTGGAGCTCGTGGCAAAGCAGAACCCAAATGTGAAGATGGGCGGCCGCTATGCCCCCAGGGACTGCGTCTCTCCTCACAAGGTGGCCATCATCATTCCATTCCGCAACCGGCAGGAGCACCTCAAGTACTGGCTATATTATTTGCACCCAGTCCTGCAGCGCCAGCAGCTGGACTATGGCATCTATGTTATCAACCAGGCGGGAGACACTATATTCAATCGTGCTAAGCTCCTCAATGTTGGCTTTCAAGAAGCCTTGAAGGACTATGACTACACCTGCTTTGTGTTTAGTGACGTGGACCTCATTCCAATGAATGACCATAATGCGTACAGGTGTTTTTCACAGCCACGGCACATTTCCGTTGCAATGGATAAGTTTGGATTCAGCCTACCTTATGTTCAGTATTTTGGAGGTGTCTCTGCTCTAAGTAAACAACAGTTTCTAACCATCAATGGATTTCCTAATAATTATTGGGGCTGGGGAGGAGAAGATGATGACATTTTTAACAGATTAGTTTTTAGAGGCATGTCTATATCTCGCCCAAATGCTGTGGTCGGGAGGTGTCGCATGATCCGCCACTCAAGAGACAAGAAAAATGAACCCAATCCTCAGAGGTTTGACCGAATTGCACACACAAAGGAGACAATGCTCTCTGATGGTTTGAACTCACTCACCTACCAGGTGCTGGATGTACAGAGATACCCATTGTATACCCAAATCACAGTGGACATCGGGACACCGAGC**TAGGGATCC**

NdeI (CATATG) and BamHI (GGATCC) restriction enzyme sites and the stop codon (TAG) to prepare a protein construct from amino acid residues 99 to 398 are indicated.

Expression of the B4GalT1 protein was done using a Ptac promoter in a modified pET23 vector as described by Gaciarz et al.

[Anna Gąciarz](https://www.ncbi.nlm.nih.gov/pubmed/?term=G%26%23x00105%3Bciarz%20A%5BAuthor%5D&cauthor=true&cauthor_uid=28619018), [Narendar Kumar Khatri](https://www.ncbi.nlm.nih.gov/pubmed/?term=Khatri%20NK%5BAuthor%5D&cauthor=true&cauthor_uid=28619018), [M. Lourdes Velez-Suberbie](https://www.ncbi.nlm.nih.gov/pubmed/?term=Velez-Suberbie%20ML%5BAuthor%5D&cauthor=true&cauthor_uid=28619018), [Mirva J. Saaranen](https://www.ncbi.nlm.nih.gov/pubmed/?term=Saaranen%20MJ%5BAuthor%5D&cauthor=true&cauthor_uid=28619018), [Yuko Uchida](https://www.ncbi.nlm.nih.gov/pubmed/?term=Uchida%20Y%5BAuthor%5D&cauthor=true&cauthor_uid=28619018), [Eli Keshavarz-Moore](https://www.ncbi.nlm.nih.gov/pubmed/?term=Keshavarz-Moore%20E%5BAuthor%5D&cauthor=true&cauthor_uid=28619018), and [Lloyd W. Ruddock](https://www.ncbi.nlm.nih.gov/pubmed/?term=Ruddock%20LW%5BAuthor%5D&cauthor=true&cauthor_uid=28619018)

Efficient soluble expression of disulfide bonded proteins in the cytoplasm of Escherichia coli in fed-batch fermentations on chemically defined minimal media

[Microb. Cell Fact](https://www.ncbi.nlm.nih.gov/pmc/articles/PMC5471842/). 16: 108. (2017)
