# Supplementary material for: The dimeric structure of wild-type human glycosyltransferase B4GalT1
Source: PLoS One. 2018 Oct 23;13(10):e0205571. doi: 10.1371/journal.pone.0205571 (PMC6198961; doi:10.1371/journal.pone.0205571)
Supplement: S2 Fig — (DOCX) [file pone.0205571.s006.docx]

**S2 Fig. Small-angle X-ray scattering (SAXS) performed on B4GalT1, allowing to locate N-termini not seen in the crystal structures.**

| **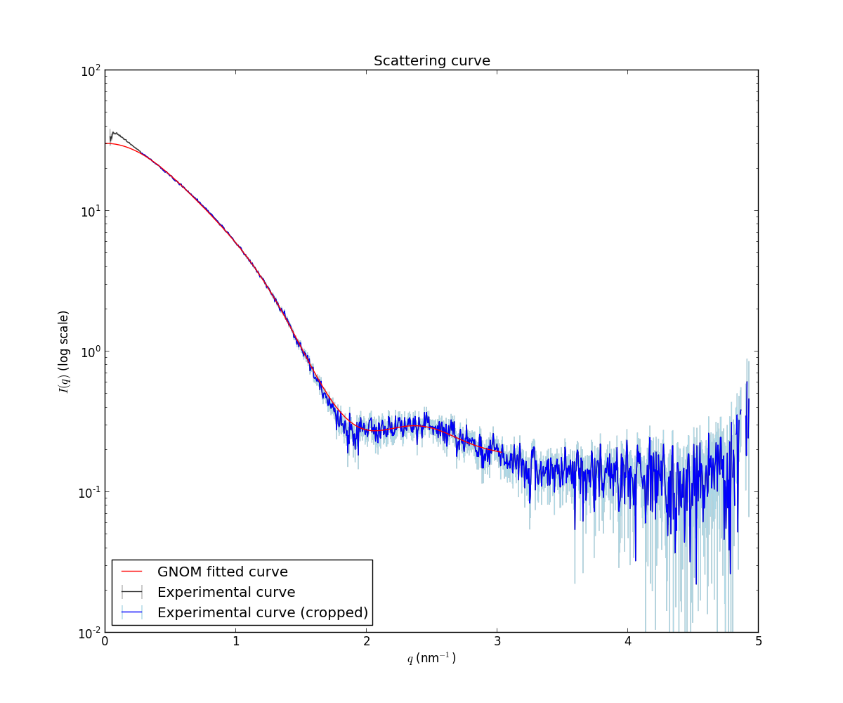** | **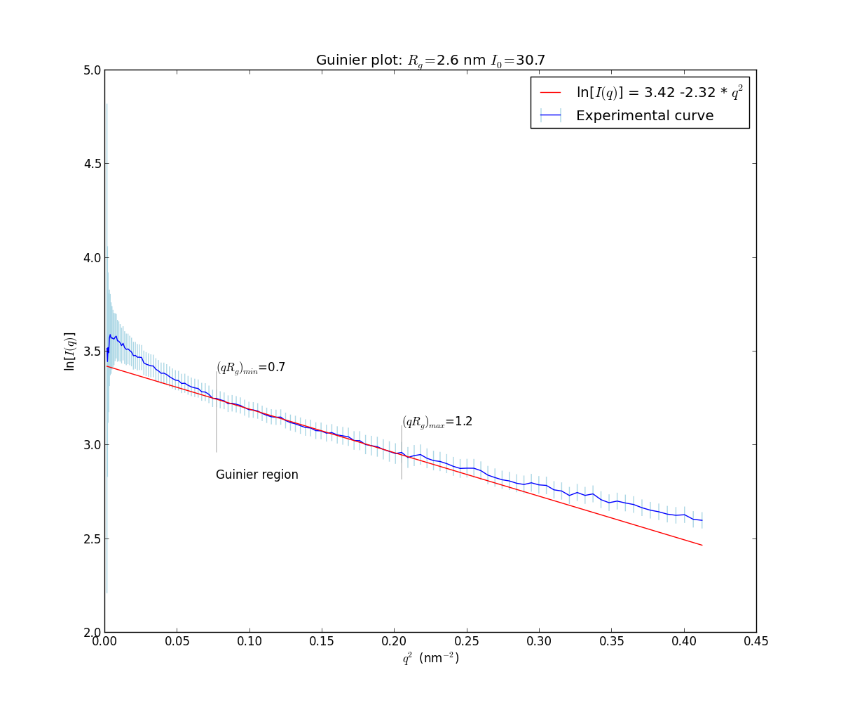** |
| --- | --- |
| **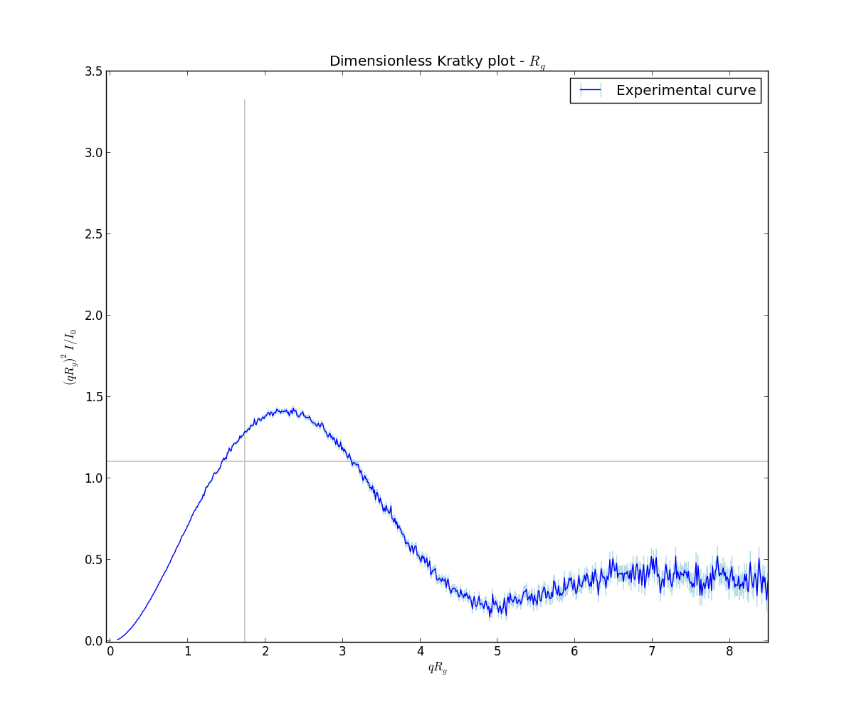** | **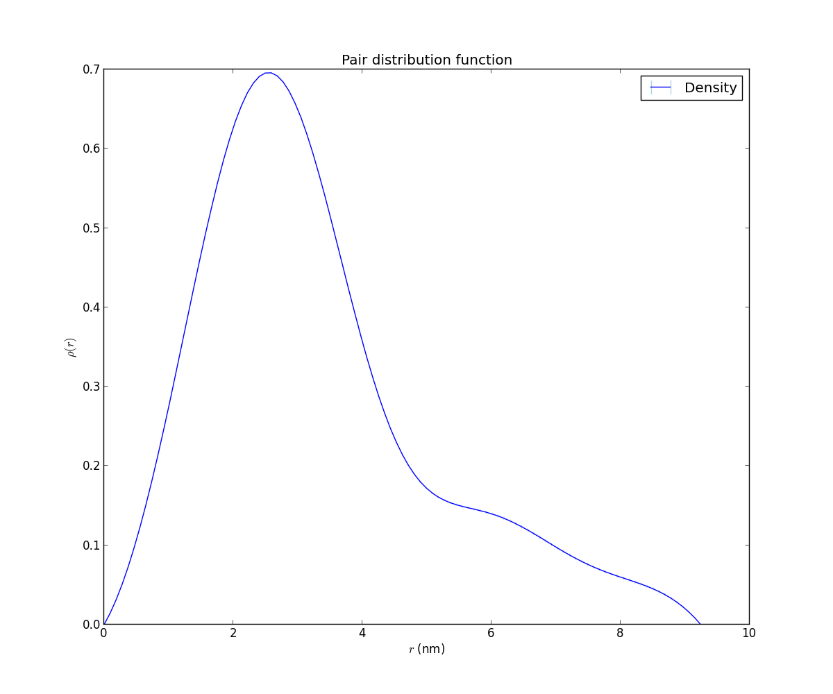** |


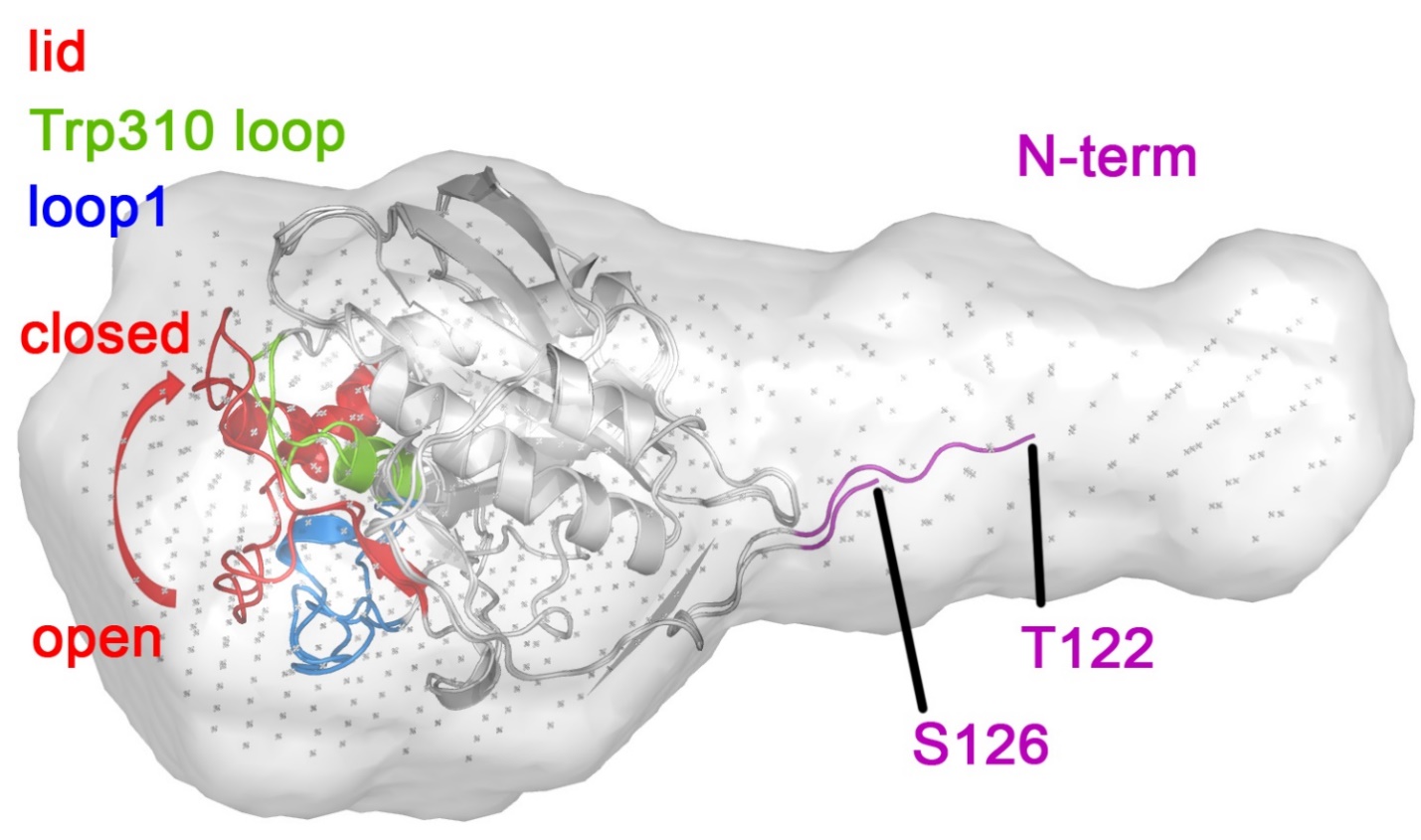


For the SAXS experiment, B4GalT1 protein was purified as for the crystallization experiments. Samples were concentrated to 1.0, 3.0, 5.0 and 7.4 mg/ml. Data were collected at beamline BM29 of the European Synchrotron Radiation Facility (Grenoble, France). The beamline setup used was calibrated with a protein solution of known mass and concentration (bovine serum albumin at 4.9 mg/ml). Data were processed using standard procedures using the program package PRIMUS (ATSAS suite). Structural parameters deduced from the treatment were estimated using the program GNOM (ATSAS suite). The radius of gyration (Rg) was estimated at 2.6 nm; The maximum particle size (Dmax) was estimated at 9.2 nm. The SAXS envelope was calculated with the DAMMIN program (ATSAS suite). The structures of open and closed B4GalT1 (6FWU and 6FWT, with the reconstructed lid) were superimposed onto the envelope with the SUPCOMB program (ATSAS suite). N-termini are indicated as purple. The B4GalT1 construct begins at D99, but the first residue seen in the crystal structure is T122 (in 6FWU) or S126 (in 6FWT). It is noticed that there are space in the envelope for the N-terminus, and that the lid side shows movement from open to closed conformation.
