# Supplementary material for: The dimeric structure of wild-type human glycosyltransferase B4GalT1
Source: PLoS One. 2018 Oct 23;13(10):e0205571. doi: 10.1371/journal.pone.0205571 (PMC6198961; doi:10.1371/journal.pone.0205571)
Supplement: S3 Fig — (DOCX) [file pone.0205571.s007.docx]

**S3 Fig. jsPISA analysis of homodimer Interface of B4GalT1 (PDB code 6FWU)**


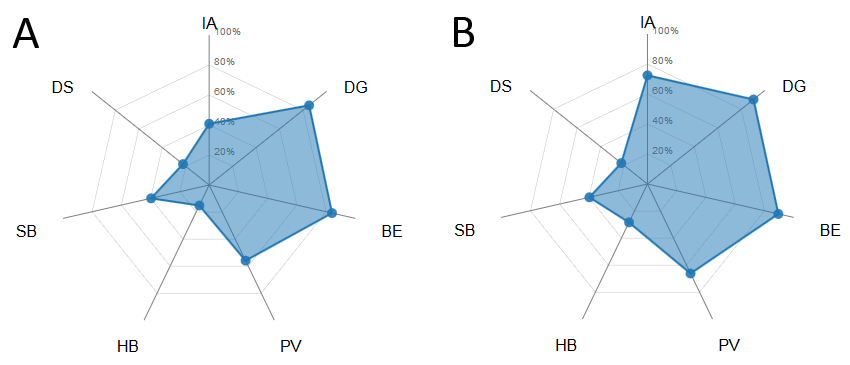


jsPISA radar for the closed conformation dimer without (A) and with (B) the reconstructed lid. IA, interface area; DG, solvation energy; BE, total binding energy; PV, hydrophobic p-value; HB, hydrogen bonds; SB, salt bridges; DS, disulphide bridges.
