# Supplementary material for: The dimeric structure of wild-type human glycosyltransferase B4GalT1
Source: PLoS One. 2018 Oct 23;13(10):e0205571. doi: 10.1371/journal.pone.0205571 (PMC6198961; doi:10.1371/journal.pone.0205571)
Supplement: S4 Fig — (DOCX) [file pone.0205571.s008.docx]

**S4 Fig.
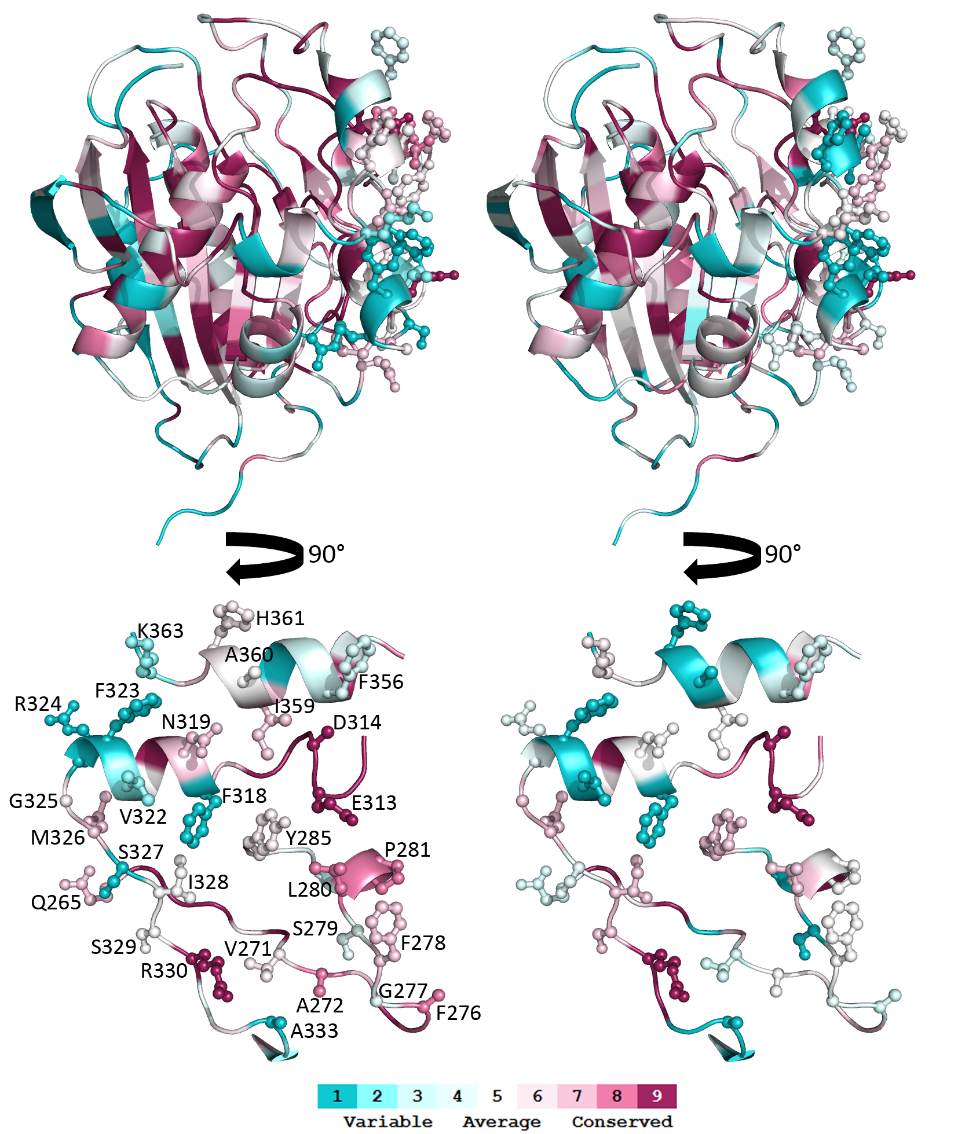
Conservation of the residues at the dimerization interface observed in the closed structure of B4GalT1 (PDB code 6FWU).**

Residues are colored according to conservation from blue to burgundy, and sticks and spheres indicate those belonging to the dimerization interface. Top: full view of one monomer from the closed dimeric B4GalT1. Bottom: highlight on the dimerization interface. Left: conservation among eukaryote species. Right: conservation among the human members of the B4GalT family (B4GalT1-7). Conservation was computed with the ConSurf webserver (http://consurf.tau.ac.il) using multiple sequence alignments presented in S5 Figure and S6 Figure (performed with Clustal Omega).
