# Supplementary material for: The dimeric structure of wild-type human glycosyltransferase B4GalT1
Source: PLoS One. 2018 Oct 23;13(10):e0205571. doi: 10.1371/journal.pone.0205571 (PMC6198961; doi:10.1371/journal.pone.0205571)
Supplement: S5 Fig — (DOCX) [file pone.0205571.s009.docx]

**S5 Fig. Multiple sequence alignment of the human members of the galactosyltransferase GT7 family**

CLUSTAL O (1.2.4) multiple sequence alignment of human members of the galactosyltransferases. The dimerization interface, as observed in the closed structure of B4GalT1, is indicated as bold on yellow background. Residues E313 and R330 of the hB4GalT’s and the corresponding residues appearing in the sequences of the other B4GalT1 enzymes are given as bold and red. The Trp loop is marked with turquoise background. The names and sequences (beginning with the gene name identifier) in the comparison are in the following order:

P15291 B4GT1_HUMAN Beta-1,4-galactosyltransferase 1

O60909 B4GT2_HUMAN Beta-1,4-galactosyltransferase 2

O60512 B4GT3_HUMAN Beta-1,4-galactosyltransferase 3

B2RAZ5 B2RAZ5_HUMAN UDP-Gal:betaGlcNAc beta 1,4-galactosyltransferase, polypeptide 4, isoform CRA_a

O43286 B4GT5_HUMAN Beta-1,4-galactosyltransferase 5

Q9UBX8 B4GT6_HUMAN Beta-1,4-galactosyltransferase 6

Q9UBV7 B4GT7_HUMAN Beta-1,4-galactosyltransferase 7

hB4GalT1 -MRLREPLL-----SGSAAMPGASLQRACRLLVAVCALHLGVTLVYYLAGRD--LSRLPQ 52

hB4GalT2 ----------------MSRLLGGTLERVCKAVLLLCLLHFLVAVILYFDVYAQHLAFFSR 44

hB4GalT3 -------------------MLRRLLERPCTLALLVGS-QLAVMMYLSLGGFRSLSALFGR 40

hB4GalT4 ----------------MGFNLTFHLSYKFRLLLL-------LTLCLTVVGWATSNYFVGA 37

hB4GalT5 -MRARRGLLRLPRRSLLAALFFFSLSSSLLYF------------VYVAPGIVNTYLFMMQ 47

hB4GalT6 -MSVLRRMMRVSNRSLLAFIFFFSLSSSCLYF------------IYVAPGIANTYLFMVQ 47

hB4GalT7 MFPSRRKAAQLPWEDGRSGLLSGGLPRKCSVFHLFVA-------CLSLGFFS-LLWLQLS 52

hB4GalT1 LVGVSTPLQGGSNSAAAI--GQSSGELRTGGARPPPPLGASSQPRPGGDSSPVVDSGPGP 110

hB4GalT2 FSA------RGPAHALHP--AASSSSSSSNC------------SRPNAT-----ASSSGL 79

hB4GalT3 DQGPTF-------DYSHP--RD----VYSN-------------------LSHL--P---- 62

hB4GalT4 IQE-----------IPKA--KE----FMAN-------------------FHKTLILGK-- 59

hB4GalT5 AQGILIR--DNVRTIGAQVYEQVLRSAYAKR---NSSVNDSDYPLDL-------NHSETF 95

hB4GalT6 ARGIMLR--ENVKTIGHMI------RLYTNK---NSTLNGTDYPEGN-------NSSDYL 89

hB4GalT7 CSG----------DVARA--------VRGQ------------------------------ 64

hB4GalT1 ASNLTSVPVPHTTALSLPACPEESPLLVGPMLIEFNMP--VDLELVAKQNPNVKMGGRYA 168

hB4GalT2 --PEVPSALPGPTAPTLPPCPDSPPGLVGRLLIEFTSP--MPLERVQRENPGVLMGGRYT 135

hB4GalT3 -----GAPGGPPAPQGLPYCPERSPLLVGPVSVSFSPV--PSLAEIVERNPRVEPGGRYR 115

hB4GalT4 --GKTLTNEASTKKVELDNCPSVSPYLRGQSKLIFKPD--LTLEEVQAENPKVS-RGRYR 114

hB4GalT5 LQTTTFLPE-DFTYFANHTCPERLPSMKGPIDINMSEIGMDYIHELFSKDPTIKLGGHWK 154

hB4GalT6 VQTTTYLPE-NFTYSPYLPCPEKLPYMRGFLNVNVSEVSFDEIHQLFSKDLDIEPGGHWR 148

hB4GalT7 ---------GQETSGPPRACPPE------------------------------PPPEHWE 85

hB4GalT1 PRDCVSPHKVAIIIPFRNRQEHLKYWLYYLHPVLQRQQLDYGIYVINQAGDTIFNRAKLL 228

hB4GalT2 PPDCTPAQTVAVIIPFRHREHHLRYWLHYLHPILRRQRLRYGVYVINQHGEDTFNRAKLL 195

hB4GalT3 PAGCEPRSRTAIIVPHRAREHHLRLLLYHLHPFLQRQQLAYGIYVIHQAGNGTFNRAKLL 175

hB4GalT4 PQECKALQRVAILVPHRNREKHLMYLLEHLHPFLQRQQLDYGIYVIHQAEGKKFNRAKLL 174

hB4GalT5 PSDCMPRWKVAILIPFRNRHEHLPVLFRHLLPMLQRQRLQFAFYVVEQVGTQPFNRAMLF 214

hB4GalT6 PKDCKPRWKVAVLIPFRNRHEHLPIFFLHLIPMLQKQRLEFAFYVIEQTGTQPFNRAMLF 208

hB4GalT7 EDASWGPHRLAVLVPFRERFEELLVFVPHMRRFLSRKKIRHHIYVLNQVDHFRFNRAALI 145

hB4GalT1 NVGFQEALK-DYDYTCFVFSDVDLIPMNDHNAYRCF**S**-**Q**PRHIS**VA**MDK**FGFSLP**YVQ**Y**F 286

hB4GalT2 NVGFLEALKEDAAYDCFIFSDVDLVPMDDRNLYRCGD-QPRHFAIAMDKFGFRLPYAGYF 254

hB4GalT3 NVGVREALR-DEEWDCLFLHDVDLLPENDHNLYVCDPRGPRHVAVAMNKFGYSLPYPQYF 234

hB4GalT4 NVGYLEALK-EENWDCFIFHDVDLVPENDFNLYKCEE-HPKHLVVGRNSTGYRLRYSGYF 232

hB4GalT5 NVGFQEAMK-DLDWDCLIFHDVDHIPESDRNYYGCGQ-MPRHFATKLDKYMYLLPYTEFF 272

hB4GalT6 NVGFKEAMK-DSVWDCVIFHDVDHLPENDRNYYGCGE-MPRHFAAKLDKYMYILPYKEFF 266

hB4GalT7 NVGFLESSN---STDYIAMHDVDLLPLNEELDYGFPEAGPFHVASP--ELHPLYHYKTYV 200

hB4GalT1 GGVSALSKQQFLTINGFPNNYWGWGG**E**DDDI**FN**RL**VFRGMSISR**PN**A**VVGRCRMIRHSRD 346

hB4GalT2 GGVSGLSKAQFLRINGFPNEYWGWGG**E**DDDIFNRISLTGMKIS**R**PDIRIGRYRMIKHDRD 314

hB4GalT3 GGVSALTPDQYLKMNGFPNEYWGWGG**E**DDDIATRVRLAGMKIS**R**PPTSVGHYKMVKHRGD 294

hB4GalT4 GGVTALSREQFFKVNGFSNNYWGWGG**E**DDDLRLRVELQRMKIS**R**PLPEVGKYTMVFHTRD 292

hB4GalT5 GGVSGLTVEQFRKINGFPNAFWGWGG**E**DDDLWNRVQNAGYSVS**R**PEGDTGKYKSIPHHH- 331

hB4GalT6 GGVSGLTVEQFRKINGFPNAFWGWGG**E**DDDLWNRVHYAGYNVT**R**PEGDLGKYKSIPHHH- 325

hB4GalT7 GGILLLSKQHYRLCNGMSNRFWGWGR**E**DDEFYRRIKGAGLQLF**R**PSGITTGYKTFRHLHD 260

hB4GalT1 KKNEPNPQR**FD**R**IAH**T**K**---ETMLSDGLNSLTYQVLDVQRYPL------YTQITVDIGTP 397

hB4GalT2 KHNEPNPQRFTKIQNTK---LTMKRDGIGSVRYQVLEVSRQPL------FTNITVDIGRP 365

hB4GalT3 KGNEENPHRFDLLVRTQ---NSWTQDGMNSLTYQLLARELGPL------YTNITADIGTD 345

hB4GalT4 KGNEVNAERMKLLHQVS---RVWRTDGLSSCSYKLVSVEHNPL------YINITVDFWFG 343

hB4GalT5 RGEVQFLGRYALLRKSK---ERQGLDGLNNLNYFA-NITYDAL------YKNITVNLTPE 381

hB4GalT6 RGEVQFLGRYKLLRYSK---ERQYIDGLNNLIYRP-KILVDRL------YTNISVNLMPE 375

hB4GalT7 PAWRKRDQKR-IAAQKQEQFKVDREGGLNTVKYHVASRTALSVGGAPCTVLNIMLDCDKT 319

hB4GalT1 S------------------------------------------------ 398

hB4GalT2 PS-WPPRG----------------------------------------- 372

hB4GalT3 PR-GPRAPSGPRYPPGSSQAFRQEMLQRRPPARPGPLSTANHTALRGSH 393

hB4GalT4 A------------------------------------------------ 344

hB4GalT5 LA-QV-----NEY------------------------------------ 388

hB4GalT6 LA-PI-----EDY------------------------------------ 382

hB4GalT7 ATPWCT------FS----------------------------------- 327
