# Supplementary material for: The dimeric structure of wild-type human glycosyltransferase B4GalT1
Source: PLoS One. 2018 Oct 23;13(10):e0205571. doi: 10.1371/journal.pone.0205571 (PMC6198961; doi:10.1371/journal.pone.0205571)
Supplement: S6 Fig — (DOCX) [file pone.0205571.s010.docx]

**S6 Fig. Multiple sequence alignment of the human members of the GT7 family**

CLUSTAL O (1.2.4) multiple sequence alignment of human glycosyltransferases of family 7, making it possible to compare hB4GalT enzymes to GTases, which use different substrates than galactose. The dimerization interface, as observed in the closed structure of B4GalT1, is indicated as bold on yellow background. Residues E313 and R330 of the hB4GalT’s and the corresponding residues appearing in the sequences of the other GT7 family enzymes are given as bold and red. The Trp loop is marked with turquoise background.

The names and sequences (beginning with the gene name identifier) in the comparison are in the following order:

P15291 B4GT1_HUMAN Beta-1,4-galactosyltransferase 1

O60909 B4GT2_HUMAN Beta-1,4-galactosyltransferase 2

O60512 B4GT3_HUMAN Beta-1,4-galactosyltransferase 3

B2RAZ5 B2RAZ5_HUMAN UDP-Gal:betaGlcNAc beta 1,4-galactosyltransferase, polypeptide 4, isoform CRA_a

O43286 B4GT5_HUMAN Beta-1,4-galactosyltransferase 5

Q9UBX8 B4GT6_HUMAN Beta-1,4-galactosyltransferase 6

Q9UBV7 B4GT7_HUMAN Beta-1,4-galactosyltransferase 7

Q6L9W6 B4GN3_HUMAN Beta-1,4-N-acetylgalactosaminyltransferase 3

Q76KP1 B4GN4_HUMAN N-acetyl-beta-glucosaminyl-glycoprotein 4-beta-N-acetylgalactosaminyltransferase 1

Q86X52 CHSS1_HUMAN Chondroitin sulfate synthase 1

Q8IZ52 CHSS2_HUMAN Chondroitin sulfate synthase 2

Q8TDX6 CGAT1_HUMAN Chondroitin sulfate N-acetylgalactosaminyltransferase 1

Q8N6G5 CGAT2_HUMAN Chondroitin sulfate N-acetylgalactosaminyltransferase 2

A2V663 A2V663_HUMAN Hexosyltransferase

G5E9W2 G5E9W2_HUMAN Hexosyltransferase

hB4GalT1 ------------------------------------------------------------ 0

hB4GalT2 ------------------------------------------------------------ 0

hB4GalT3 ------------------------------------------------------------ 0

hB4GalT4 ------------------------------------------------------------ 0

hB4GalT5 ------------------------------------------------------------ 0

hB4GalT6 ------------------------------------------------------------ 0

hB4GalT7 ------------------------------------------------------------ 0

hB4GalNAcT3 MGSPRAARPPLLLRPVKLLRRRFRLLLA-LAVVSVGLWTLYLELVASAQV--GGNPLNRR 57

hB4GalNAcT4 ----------MPRLPVKKIRKQMKLLLL-LLLLSCAAWLTYVHLGLVRQ----GRALRQR 45

hCHSY1 ----------M---AARGRRAWLSVLLGLVLGFVLASRLVLPRASELKRAGPRRR----- 42

hCHSY2 ------MRASL---LLSVLRPAGPVAVGISLGFTL------------------------- 26

hCHGN1 ------------------------------------------------------------ 0

hCHGN2 ------------------------------------------------------------ 0

hCHPF1 ----------M---AVRSRRPWMSVALGLVLGFTAASWLIAPRVAELSERKRRGSSLCSY 47

hCHPF2 --------------MLSLARPP---LPPTGLRTSL------------------------- 18

hB4GalT1 ------------------------------------------------------------ 0

hB4GalT2 ------------------------------------------------------------ 0

hB4GalT3 ------------------------------------------------------------ 0

hB4GalT4 ------------------------------------------------------------ 0

hB4GalT5 ------------------------------------------------------------ 0

hB4GalT6 ------------------------------------------------------------ 0

hB4GalT7 ------------------------------------------------------------ 0

hB4GalNAcT3 YGSWRELAKAL---ASRNIPAVDPHLQFYHPQ------------RLSLEDHDIDQGVS-- 100

hB4GalNAcT4 LGYGRDGEKLTSETDGRGVHAA-PSTQRAEDSSESREE------EQAPEGRDLDMLFPGG 98

hCHSY1 --------------------------------------ASP------------------- 45

hCHSY2 ------------------------------------------------------------ 26

hCHGN1 ------------------------------------------------------------ 0

hCHGN2 ------------------------------------------------------------ 0

hCHPF1 YGRSAAGPRA-----GAQQPLP-------QPQSRPRQEQSPPPARQDLQGPPLPEAAPGI 95

hCHPF2 ------------------------------------------------------------ 18

hB4GalT1 ------------------------------------------------------------ 0

hB4GalT2 ------------------------------------------------------------ 0

hB4GalT3 ------------------------------------------------------------ 0

hB4GalT4 ------------------------------------------------------------ 0

hB4GalT5 ------------------------------------------------------------ 0

hB4GalT6 ------------------------------------------------------------ 0

hB4GalT7 ------------------------------------------------------------ 0

hB4GalNAcT3 -SNSSYLKWNKPVPWLSEFRGRANLHVFEDWCGSSIQQLRRNLHFPLYPHIRTTLRKLAV 159

hB4GalNAcT4 AGRLPLNFTHQTPPWREEYKGQVNLHVFEDWCGGAVGHLRRNLHFPLFPHTRTTVKKLAV 158

hCHSY1 -----------------------------EGCRSGQA----------------------- 53

hCHSY2 --SLLSVTWVEE------------------PCGPGPPQPGDSE-LP--PRGNTNA---AR 60

hCHGN1 ------------------------------------------------------------ 0

hCHGN2 ------------------------------------------------------------ 0

hCHPF1 -TSFRSSPWQQPPPLQQRRRGRE-----PEGATGLPGAPAAE-----------------G 132

hCHPF2 --SLLRVSWIQGEGE--------------DPCVEAVGERGGP-----------------Q 45

hB4GalT1 ------------------------------------------------------------ 0

hB4GalT2 ------------------------------------------------------------ 0

hB4GalT3 ------------------------------------------------------------ 0

hB4GalT4 ------------------------------------------------------------ 0

hB4GalT5 ------------------------------------------------------------ 0

hB4GalT6 ------------------------------------------------------------ 0

hB4GalT7 ------------------------------------------------------------ 0

hB4GalNAcT3 SPKWTNYGLRIFGYLHPFTDGKIQFAIAADDNAEFWLSLDDQVSGLQLLASVGKTGKEWT 219

hB4GalNAcT4 SPKWKNYGLRIFGFIHPARDGDVQFSVASDDNSEFWLSLDESPAAAQLVAFVGKTGSEWT 218

hCHSY1 -AAS-------------------------------------------------------Q 57

hCHSY2 RPNS-------------------------------------------------------V 65

hCHGN1 ------------------------------------------------------------ 0

hCHGN2 ------------------------------------------------------------ 0

hCHPF1 EPEE-------------------------------------------------------E 137

hCHPF2 NPD--------------------------------------------------------- 48

hB4GalT1 ------------------------------------------------------------ 0

hB4GalT2 ------------------------------------------------------------ 0

hB4GalT3 ------------------------------------------------------------ 0

hB4GalT4 ------------------------------------------------------------ 0

hB4GalT5 ------------------------------------------------------------ 0

hB4GalT6 ------------------------------------------------------------ 0

hB4GalT7 ------------------------------------------------------------ 0

hB4GalNAcT3 APGEFGKFRSQISKPVSLSASHRYYFEVLHKQNEEGT-------DHVEVAWRRNDP---- 268

hB4GalNAcT4 APGEFTKFSSQVSKPRRLMASRRYYFELLHKQDDRGS-------DHVEVGWRAFLP---- 267

hCHSY1 AGGARG--------------------------DARG--------------AQ----LW-- 71

hCHSY2 QPGAER--------------------------EKPG------AGEGAGENWEPRVLPYHP 93

hCHGN1 ------------------------------------------------------------ 0

hCHGN2 ------------------------------------------------------------ 0

hCHPF1 DGGAAG--------------------------QRRD--------------GRPGSSHNG- 156

hCHPF2 ---------------------------------SRA------RLDQSDEDFKPRIVPYYR 69

hB4GalT1 ------------------------------------------------------------ 0

hB4GalT2 ------------------------------------------------------------ 0

hB4GalT3 ------------------------------------------------------------ 0

hB4GalT4 ------------------------------------------------------------ 0

hB4GalT5 ------------------------------------------------------------ 0

hB4GalT6 ------------------------------------------------------------ 0

hB4GalT7 ------------------------------------------------------------ 0

hB4GalNAcT3 ---GAKF-TI------------IDSLSLSLFTNETFLQMDEVGH-------IPQTAASHV 305

hB4GalNAcT4 ---GLKF-EV------------ISSAHISLYTDESALKMDHVAH-------VPQSPASHV 304

hCHSY1 -PPGSDP---------DGGPRDRNFLFVGVMTAQKYLQTRAVAAYRTWSKTIPGK--VQF 119

hCHSY2 AQPGQAAKKAVRTRYISTELGIRQRLLVAVLTSQTTLPTLGVAVNRTLGHRLERV--VFL 151

hCHGN1 ------------------------------------------------------------ 0

hCHGN2 ------------------------------------------------------------ 0

hCHPF1 SGDGGAA---------APSARPRDFPYVGVMTAQKYLGSRALAAQRTRARFIPGH--VEF 205

hCHPF2 -DPNKPYKKVLRTRYIQTELGSRERLLVAVLTSRATLSTLAVAVNRTVAHHFPRL--LYF 126

hB4GalT1 ------------------------------------------------------------ 0

hB4GalT2 ------------------------------------------------------------ 0

hB4GalT3 ------------------------------------------------------------ 0

hB4GalT4 ------------------------------------------------------------ 0

hB4GalT5 ------------------------------------------------------------ 0

hB4GalT6 ------------------------------------------------------------ 0

hB4GalT7 ------------------------------------------------------------ 0

hB4GalNAcT3 DSSNALPRDEQPPADM--LRPDPRDTLYRVPLIPKSHLRHVLPDCPYKPSYLVDGLPLQR 363

hB4GalNAcT4 GG---RPPQEETSADM--LRPDPRDTFFLTPRMESSSLENVLEPCAYAPTYVVKDFPIAR 359

hCHSY1 FSSEGS----DTSVPIPVVPLRGVDDSYPP----------------------------QK 147

hCHSY2 TGARGR----RAPPGMAVVTLG---EE-RP----------------------------IG 175

hCHGN1 ------------------------------------------------------------ 0

hCHGN2 ------------------------------------------------------------ 0

hCHPF1 FSSQQPPNAGQPPPPLPVIALPGVDDSYPP----------------------------QK 237

hCHPF2 TGQRGA----RAPAGMQVVSHG---DE-RP----------------------------AW 150

hB4GalT1 ------------------------------------------------------------ 0

hB4GalT2 ------------------------------------------------------------ 0

hB4GalT3 ------------------------------------------------------------ 0

hB4GalT4 ------------------------------------------------------------ 0

hB4GalT5 ------------------------------------------------------------ 0

hB4GalT6 ------------------------------------------------------------ 0

hB4GalT7 ------------------------------------------------------------ 0

hB4GalNAcT3 YQGLRFVHLSFVYPNDYTRLSHMETHNKCFYQENAYYQDRFSFQEYI----------KID 413

hB4GalNAcT4 YQGLQFVYLSFVYPNDYTRLTHMETDNKCFYRESPLYLERFGFYKYM----------KMD 409

hCHSY1 KSFMMLKYMHDHYLDKYEWFMRA--DDDVYIK---------------------------- 177

hCHSY2 HLHLALRHLLEQHGDDFDWFFLV--PDTTYTE---------------------------- 205

hCHGN1 ------------------------------------------------------------ 0

hCHGN2 ------------------------------------------------------------ 0

hCHPF1 KSFMMIKYMHDHYLDKYEWFMRA--DDDVYIK---------------------------- 267

hCHPF2 LMSETLRHLHTHFGADYDWFFIM--QDDTYVQ---------------------------- 180

hB4GalT1 ------------------------------------------------------------ 0

hB4GalT2 ------------------------------------------------------------ 0

hB4GalT3 ------------------------------------------------------------ 0

hB4GalT4 ------------------------------------------------------------ 0

hB4GalT5 ------------------------------------------------------------ 0

hB4GalT6 ------------------------------------------------------------ 0

hB4GalT7 ------------------------------------------------------------ 0

hB4GalNAcT3 QPEKQGLEQPGFEE---------NLLEESQYGEVAEETPASNN-QNARMLEGRQTPAST- 462

hB4GalNAcT4 KEEGDEDEEDEVQRRAFLFLNPDDFLDDEDEGELLDSLEPTEA---APPRSGPQSPAPA- 465

hCHSY1 -----GDRLENFLR--SLNSSEPLFLGQTGLGTTEEMGKLALEPGENFCMGGPGVIMSRE 230

hCHSY2 -----AHGLARLTGHLSLASAAHLYLGRPQ-----DFIGGEP-TPGRYCHGGFGVLLSRM 254

hCHGN1 -----------------------------------------------MMMVRRGLLAWIS 13

hCHGN2 -------------------------------------------------MPRRGLILHTR 11

hCHPF1 -----EDKLEEFLR--SLNSSKPLYLGQTGLGNIEELGKLGLEPGENFCMGGPGMIFSRE 320

hCHPF2 -----APRLAALAGHLSIN--QDLYLGRAE-----EFIGAG--EQARYCHGGFGYLLSRS 226

hB4GalT1 ------------------------------------------------------------ 0

hB4GalT2 ------------------------------------------------------------ 0

hB4GalT3 ------------------------------------------------------------ 0

hB4GalT4 ------------------------------------------------------------ 0

hB4GalT5 ------------------------------------------------------------ 0

hB4GalT6 ------------------------------------------------------------ 0

hB4GalT7 ------------------------------------------------------------ 0

hB4GalNAcT3 ------------------------------------------------------------ 462

hB4GalNAcT4 ------------------------------------------------------------ 465

hCHSY1 VLRRMVPHIGKC--LREMYTTHEDVEVGRCVRRFAGVQCVWSYEMQQLFYENYEQNKKGY 288

hCHSY2 LLQQLRPHLEGC--RNDIVSARPDEWLGRCILDATGVGCTGDHEGVHYSH--LELSP-GE 309

hCHGN1 R--VVVLLVLLCCAISVLYM-------LACTPKGDEEQLALPR-------ANSPTGKEGY 57

hCHGN2 THWLLLGLALLCSLVLFMYL-------LECAPQTDGNASLPGV-------VGENYGKEYY 57

hCHPF1 VLRRMVPHIGEC--LREMYTTHEDVEVGRCVRRFGGTQCVWSYEMQQLFHENYEHNRKGY 378

hCHPF2 LLLRLRPHLDGC--RGDILSARPDEWLGRCLIDSLGVGCVSQHQGQQYRS--FELAKNRD 282

hB4GalT1 ------------------------------------------------------------ 0

hB4GalT2 ------------------------------------------------------------ 0

hB4GalT3 ------------------------------------------------------------ 0

hB4GalT4 ------------------------------------------------------------ 0

hB4GalT5 ------------------------------------------------------------ 0

hB4GalT6 ------------------------------------------------------------ 0

hB4GalT7 ------------------------------------------------------------ 0

hB4GalNAcT3 -----------L------EQDATDYRLRSLRKLL--AQPREGLL-------APF-----S 491

hB4GalNAcT4 -----------A------PAQPGA-TL--APPTP--PRPRDGGT-------PRH-----S 491

hCHSY1 IRDLHNSKIHQAITLHPNKNPPYQYRLHSYMLSRKISELRHRT----------------- 331

hCHSY2 PVQEGDPHFRSALTAHPVRDPVHMYQLHKAFARAELERTYQEIQ---------------- 353

hCHGN1 QAVLQEWEE--------------QHRNYVSSLKRQIAQLKEELQ---------------- 87

hCHGN2 QALLQEQEE--------------HYQTRATSLKRQIAQLKQELQ---------------- 87

hCHPF1 IQDLHNSKIHAAITLHPNKRPAYQYRLHNYMLSRKISELRYRT----------------- 421

hCHPF2 PEKEGSSAFLSAFAVHPVSEGTLMYRLHKRFSALELERAYSEIE---------------- 326

hB4GalT1 ------------------------------------------------------------ 0

hB4GalT2 ------------------------------------------------------------ 0

hB4GalT3 ------------------------------------------------------------ 0

hB4GalT4 ------------------------------------------------------------ 0

hB4GalT5 ------------------------------------------------------------ 0

hB4GalT6 ------------------------------------------------------------ 0

hB4GalT7 ------------------------------------------------------------ 0

hB4GalNAcT3 KRN--------STAS--FPGRTSH--IPVQQPEK------------R----KQKPSPEP- 522

hB4GalNAcT4 RALSWAARAARPLPL--FLGRAPPPRPAVEQPPP------------KVYVTRVRPGQRA- 536

hCHSY1 ---------IQLHRE-----------------IVLMSKYSNTEIHKEDLQLGIPPS--FM 363

hCHSY2 -ELQWEIQNTSHLAVDGD--------------------------QAAAWPVGIPAP---- 382

hCHGN1 -ERSEQLRNGQYQAS-DAAGLGLD-RSPPEKTQADLLAFLHSQVDKAEVNAGVKLA---- 140

hCHGN2 -EMSEKMRSLQERRNVGANGIGYQ--SNKEQAPSDLLEFLHSQIDKAEVSIGAKLP---- 140

hCHPF1 ---------IQLHRE-----------------SALMSKLSNTEVSKEDQQLGVIPS--FN 453

hCHPF2 -QLQAQIRNLTVLTPEGE--------------------------AGLSWPVGLPAP---- 355

hB4GalT1 ------------------------------------------------------------ 0

hB4GalT2 ------------------------------------------------------------ 0

hB4GalT3 ------------------------------------------------------------ 0

hB4GalT4 ------------------------------------------------------------ 0

hB4GalT5 ------------------------------------------------------------ 0

hB4GalT6 ------------------------------------------------------------ 0

hB4GalT7 ------------------------------------------------------------ 0

hB4GalNAcT3 --S-QDSPHSDKWPPGHPVKNL----PQ--MRGP-R-P-RPAGDSPRKTQW--------- 561

hB4GalNAcT4 --SPRAPAPRAPWPPFPGVFLHPRPLPRVQLRAPPRPP-RPHGRRTGGPQATQPRPPARA 593

hCHSY1 RFQPRQREEILEWEFLTGKYLYS-----AVDGQPPRRGMDSAQRE--------------- 403

hCHSY2 -SRPASRFEVLRWDYFTEQHAFSC--ADGS----PRCPLRGADRA--------------- 420

hCHGN1 -----TEYAAVPFDSFTLQKVYQL--ETGLTRHPEEKPVRKDKRD--------------- 178

hCHGN2 -----SEYGVIPFESFTLMKVFQL--EMGLTRHPEEKPVRKDKRD--------------- 178

hCHPF1 HFQPRERNEVIEWEFLTGKLLYS-----AAENQPPRQSLSSILRT--------------- 493

hCHPF2 -FTPHSRFEVLGWDYFTEQHTFSC--ADGA----PKCPLQGASRA--------------- 393

hB4GalT1 ------------------------------------------------------------ 0

hB4GalT2 ------------------------------------------------------------ 0

hB4GalT3 ------------------------------------------------------------ 0

hB4GalT4 ------------------------------------------------------------ 0

hB4GalT5 ------------------------------------------------------------ 0

hB4GalT6 ------------------------------------------------------------ 0

hB4GalT7 ------------------------------------------------------------ 0

hB4GalNAcT3 ----------------LN----QVESYIAEQ--------RRGDRMRPQAPGRGWHGEEEV 593

hB4GalNAcT4 QATQG---GREGQARTLGPAAPTVDSNLSSEARPVTSFLSLSQVSGPQLPGEGEEEEE-- 648

hCHSY1 -----------ALDDIVM----QVMEMINAN-----------AKTRG------------- 424

hCHSY2 -----------DVADVLG----TALEELNRR-----------YHPA-------------- 440

hCHGN1 -----------ELVEAIE----SALETLNSP-----------AENSP------------- 199

hCHGN2 -----------ELVEVIE----AGLEVINNP-----------DEDDE------------- 199

hCHPF1 -----------ALDDTVL----QVMEMINEN-----------AKSRG------------- 514

hCHPF2 -----------DVGDALE----TALEQLNRR-----------YQPR-------------- 413

hB4GalT1 -----------------------------------------------MRLREPLL----- 8

hB4GalT2 ------------------------------------------------------------ 0

hB4GalT3 ------------------------------------------------------------ 0

hB4GalT4 ------------------------------------------------------------ 0

hB4GalT5 -----------------------------------------------MRARRGLLRLPRR 13

hB4GalT6 -----------------------------------------------MSVLRRMMRVSNR 13

hB4GalT7 ----------------------------------------------MFPSRRKAAQLPWE 14

hB4GalNAcT3 VAAAGQEGQVEGEEEGEEEEEEEDMSEVFEYVPVFDPVVNWDQTFSARNLDFQALRTDWI 653

hB4GalNAcT4 ----GEDDGAPGDEAASEDSEE---AAGPALGRWREDAIDWQRTFSVGAVDFELLRSDWN 701

hCHSY1 ------------------------------------------------------------ 424

hCHSY2 ------------------------------------------------------------ 440

hCHGN1 ----------------N------------------------------------------- 200

hCHGN2 ----------------QEDEEG---PL--------------------------------- 207

hCHPF1 ------------------------------------------------------------ 514

hCHPF2 ------------------------------------------------------------ 413

hB4GalT1 SGSAAMPGASLQRACRLLVAVCALHLGVTLVYYLAGRD--LSRLPQLVGVSTPLQGGSNS 66

hB4GalT2 --MSRLLGGTLERVCKAVLLLCLLHFLVAVILYFDVYAQHLAFFSRFSA------RGPAH 52

hB4GalT3 -----MLRRLLERPCTLALLVGS-QLAVMMYLSLGGFRSLSALFGRDQGPTF-------D 47

hB4GalT4 --MGFNLTFHLSYKFRLLLL-------LTLCLTVVGWATSNYFVGAIQE----------- 40

hB4GalT5 SLLAALFFFSLSSSLLYF------------VYVAPGIVNTYLFMMQAQGILIR--DNVRT 59

hB4GalT6 SLLAFIFFFSLSSSCLYF------------IYVAPGIANTYLFMVQARGIMLR--ENVKT 59

hB4GalT7 DGRSGLLSGGLPRKCSVFHLFVA-------CLSLGFFS-LLWLQLSCSG----------D 56

hB4GalNAcT3 DLSCNTSG-NLLLPEQEA-------LEVTRVF--------LKKLNQRSRGRYQLQRIVNV 697

hB4GalNAcT4 DLRCNVSG-NLQLPEAEA-------VDVTAQY--------MERLNARHGGRFALLRIVNV 745

hCHSY1 -----------------------------RIIDFKEIQYGYRRVNPMYGAEYILDLLLLY 455

hCHSY2 -----------------------------LRLQKQQLVNGYRRFDPARGMEYTLDLQLEA 471

hCHGN1 ----------------------------HRPYTASDFIEGIYRTERDKGTLYELTF---- 228

hCHGN2 --------------------------GEKLIFNENDFVEGYYRTERDKGTQYELFF---- 237

hCHPF1 -----------------------------RLIDFKEIQYGYRRVNPMHGVEYILDLLLLY 545

hCHPF2 -----------------------------LRFQKQRLLNGYRRFDPARGMEYTLDLLLEC 444

hB4GalT1 AAAI--GQSSGELRTGGARPPPPLGASSQPRPGGDSSPVVDSGPGPASNLT-----SVPV 119

hB4GalT2 ALHP--AASSSSSSSNC------------SRPNAT-----ASSSGL--PEV-----PSAL 86

hB4GalT3 YSHP--RD----VYSN-------------------LSHL--P--------------GAPG 66

hB4GalT4 IPKA--KE----FMAN-------------------FHKTLILGK----GKT-----LTNE 66

hB4GalT5 IGAQVYEQVLRSAYAKR---NSSVNDSDYPLDL-------NHSETFLQTTT-----FLPE 104

hB4GalT6 IGHMI------RLYTNK---NSTLNGTDYPEGN-------NSSDYLVQTTT-----YLPE 98

hB4GalT7 VARA--------VRGQ-------------------------------------------- 64

hB4GalNAcT3 EKRQ------DQLRGG-------------------------------------------- 707

hB4GalNAcT4 EKRR------DSARGS-------------------------------------------- 755

hCHSY1 KKHK----------GKK--MTVPVRRHAYLQQT--F-----SKIQFVEHEELDAQELAKR 496

hCHSY2 LTPQ----------GGR--RPLTR------------------------------------ 483

hCHGN1 KGDH---------------------KHEFK------------------------------ 237

hCHGN2 KKAD---------------------LTEYR------------------------------ 246

hCHPF1 KRHK----------GRK--LTVPVRRHAYLQQL--F-----SKPFFRETEELDVNSLVES 586

hCHPF2 VTQR----------GHR--RALAR------------------------------------ 456

hB4GalT1 PHTTALSLPACPEESPLLVGPMLIEFNMP--VDL----------------------ELVA 155

hB4GalT2 PGPTAPTLPPCPDSPPGLVGRLLIEFTSP--MPL----------------------ERVQ 122

hB4GalT3 GPPAPQGLPYCPERSPLLVGPVSVSFSPV--PSL----------------------AEIV 102

hB4GalT4 ASTKKVELDNCPSVSPYLRGQSKLIFKPD--LTL----------------------EEVQ 102

hB4GalT5 -DFTYFANHTCPERLPSMKGPIDINMSEIGMDYI----------------------HELF 141

hB4GalT6 -NFTYSPYLPCPEKLPYMRGFLNVNVSEVSFDEI----------------------HQLF 135

hB4GalT7 GQETSGPPRACPPE---------------------------------------------- 78

hB4GalNAcT3 --------RYLLELELLEQGQRVVRLSEYVSARGWQG-IDPAGGEEVEARNLQGLVWDPH 758

hB4GalNAcT4 --------RFLLELELQERGGGRLRLSEYVFLRLPGARVGDADGESPEPA---------- 797

hCHSY1 INQESG--------SLSFLSNSLKKLVPFQ------------------------------ 518

hCHSY2 ---------------------RVQLLRPLS------------------------------ 492

hCHGN1 ---------------------RLILFRPFG------------------------------ 246

hCHGN2 ---------------------HVTLFRPFG------------------------------ 255

hCHPF1 INSETQ--------SFSFISNSLKILSSFQ------------------------------ 608

hCHPF2 ---------------------RVSLLRPLS------------------------------ 465

hB4GalT1 KQNPNVKMGGRYAPRDCVSPH-------KVAIIIPFR-NRQEHLKYWLYYLHPVLQRQQ- 206

hB4GalT2 RENPGVLMGGRYTPPDCTPAQ-------TVAVIIPFR-HREHHLRYWLHYLHPILRRQR- 173

hB4GalT3 ERNPRVEPGGRYRPAGCEPRS-------RTAIIVPHR-AREHHLRLLLYHLHPFLQRQQ- 153

hB4GalT4 AENPKVS-RGRYRPQECKALQ-------RVAILVPHR-NREKHLMYLLEHLHPFLQRQQ- 152

hB4GalT5 SKDPTIKLGGHWKPSDCMPRW-------KVAILIPFR-NRHEHLPVLFRHLLPMLQRQR- 192

hB4GalT6 SKDLDIEPGGHWRPKDCKPRW-------KVAVLIPFR-NRHEHLPIFFLHLIPMLQKQR- 186

hB4GalT7 ------PPPEHWEEDASWGPH-------RLAVLVPFR-ERFEELLVFVPHMRRFLSRKK- 123

hB4GalNAcT3 NRRRQVLNTRAQEPKLCWPQGFSWSHRAVVHFVVPVK-NQARWVQQFIKDMENLFQVTGD 817

hB4GalNAcT4 ---PAASVRPDGRPELCRPLRLAWRQDVMVHFIVPVK-NQARWVAQFLADMAALHARTGD 853

hCHSY1 --LP-GSKSEHK-----------EPKDKKINILIPLS-GRFDMFVRFM---GNFEKTCLI 560

hCHSY2 -RVE-ILP-VPY-----------VTEASRLTVLLPLAAAERDLAPGFL---EAFATAALE 535

hCHGN1 -PIM-KVKNEKL-----------NMANTLINVIVPLA-KRVDKFRQFM---QNFREMCIE 289

hCHGN2 -PLM-KVKSEMI-----------DITRSIINIIVPLA-ERTEAFVQFM---QNFRDVCIH 298

hCHPF1 --GA-KEMG--------------GHNEKKVHILVPLI-GRYDIFLRFM---ENFENMCLI 647

hCHPF2 -RVE-ILP-MPY-----------VTEATRVQLVLPLLVAEAAAAPAFL---EAFAANVLE 508

hB4GalT1 -----LDYGIYVINQAG----------------------------------------DTI 221

hB4GalT2 -----LRYGVYVINQHG----------------------------------------EDT 188

hB4GalT3 -----LAYGIYVIHQAG----------------------------------------NGT 168

hB4GalT4 -----LDYGIYVIHQAE----------------------------------------GKK 167

hB4GalT5 -----LQFAFYVVEQVG----------------------------------------TQP 207

hB4GalT6 -----LEFAFYVIEQTG----------------------------------------TQP 201

hB4GalT7 -----IRHHIYVLNQVD----------------------------------------HFR 138

hB4GalNAcT3 PHFNIV---------------------ITDYSSED--MDVEMALKRSKLRSYQYVKLSGN 854

hB4GalNAcT4 SRFSVV---------------------LVDFESED--MDVERALRAARLPRYQYLRRTGN 890

hCHSY1 PNQNVKLVVLLF--NSDSNPDKAK------QV-----ELMRDYRIKYPKADMQILPVSGE 607

hCHSY2 PGDAAAALTLLLLYEPRQAQRVAHADVFAPVK-----AHVAELERRFPGARVPWLSVQTA 590

hCHGN1 QDGRVHLTVVYF--GKEEINEVKG------IL-----E---NTSKAANFRNFTFIQLNGE 333

hCHGN2 QDKKIHLTVVYF--GKEGLSKVKS------IL-----E---SVTSESNFHNYTLVSLNEE 342

hCHPF1 PKQNVKLVIILF--SRDSGQDSSK------HI-----ELIKGYQNKYPKAEMTLIPMKGE 694

hCHPF2 PREH-ALLTLLLVYGPREGGRGA-PDPFLGVK-----AAAAELERRYPGTRLAWLAVRAE 561

hB4GalT1 FNRAKLLNVGFQEALK-DYDYTCF----------------------VFSDVDLIPMNDH- 257

hB4GalT2 FNRAKLLNVGFLEALKEDAAYDCF----------------------IFSDVDLVPMDDR- 225

hB4GalT3 FNRAKLLNVGVREALR-DEEWDCL----------------------FLHDVDLLPENDH- 204

hB4GalT4 FNRAKLLNVGYLEALK-EENWDCF----------------------IFHDVDLVPENDF- 203

hB4GalT5 FNRAMLFNVGFQEAMK-DLDWDCL----------------------IFHDVDHIPESDR- 243

hB4GalT6 FNRAMLFNVGFKEAMK-DSVWDCV----------------------IFHDVDHLPENDR- 237

hB4GalT7 FNRAALINVGFLESSN---STDYI----------------------AMHDVDLLPLNEE- 172

hB4GalNAcT3 FERSAGLQAGIDL-VK---DPHSIIFLCDLHIHFPAGVIDAIRKHCVEGKMAFAPMVMR- 909

hB4GalNAcT4 FERSAGLQAGVDA-VE---DASSIVFLCDLHIHFPPNILDGIRKHCVEGRLAFAPVVMR- 945

hCHSY1 FSRALALEVGSSQ----FNN-ESLLFFCDVDLVFTTEFLQRCRANTVLGQQIYFPIIFSQ 662

hCHSY2 APSPLRLMDLLSK----KHPLDTLFLLAGPDTVLTPDFLNRCRMHAISGWQAFFPMHFQA 646

hCHGN1 FSRGKGLDVGARF----WKGSNVLLFFCDVDIYFTSEFLNTCRLNTQPGKKVFYPVLFSQ 389

hCHGN2 FNRGRGLNVGARA----WDKGEVLMFFCDVDIYFSAEFLNSCRLNAEPGKKVFYPVVFSL 398

hCHPF1 FSRGLGLEMASAQ----FDN-DTLLLFCDVDLIFREDFLQRCRDNTIQGQQVYYPIIFSQ 749

hCHPF2 APSQVRLMDVVSK----KHPVDTLFFLTTVWTRPGPEVLNRCRMNAISGWQAFFPVHFQE 617

hB4GalT1 ----NAYRCF**S**---------------------**Q**PRHIS**VA**MDK**FGFSLP**YVQ**Y**FGGVSAL 292

hB4GalT2 ----NLYRCGD---------------------QPRHFAIAMDKFGFRLPYAGYFGGVSGL 260

hB4GalT3 ----NLYVCDPR--------------------GPRHVAVAMNKFGYSLPYPQYFGGVSAL 240

hB4GalT4 ----NLYKCEE---------------------HPKHLVVGRNSTGYRLRYSGYFGGVTAL 238

hB4GalT5 ----NYYGCGQ---------------------MPRHFATKLDKYMYLLPYTEFFGGVSGL 278

hB4GalT6 ----NYYGCGE---------------------MPRHFAAKLDKYMYILPYKEFFGGVSGL 272

hB4GalT7 ----LDYGFPEA--------------------GPFHVASP--ELHPLYHYKTYVGGILLL 206

hB4GalNAcT3 ----LHCGATPQ--------------------WP-EGYWEVNGFGLLGIYKSDLDRIGGM 944

hB4GalNAcT4 ----LSCGSSPR--------------------DP-HGYWEVNGFGLFGIYKSDFDRVGGM 980

hCHSY1 YDPKIVYSG---KVP----------SDNHFAFTQKTGFWRNYGFGITCIYKGDLVRVGGF 709

hCHSY2 FHPAVAPPQGP-GPPEL---------------GRDTGRFDRQAASEACFYNSDYVAARGR 690

hCHGN1 YNPGIIYGHHDAVPP----------LEQQLVIKKETGFWRDFGFGMTCQYRSDFINIGGF 439

hCHGN2 YNPAIVYANQEVPPP----------VEQQLVHKKDSGFWRDFGFGMTCQYRSDFLTIGGF 448

hCHPF1 YDPKVTNGG---NPP----------TDDYFIFSKKTGFWRDYGYGITCIYKSDLLGAGGF 796

hCHPF2 FNPALSPQRSPPGPPGAGPDPPSPPGADPSRGAPIGGRFDRQASAEGCFYNADYLAARAR 677

hB4GalT1 SKQQFLTINGFPNNYWGWGG**E**DDDI**FN**RL**V**-**FRGMSISR**PN**A**VVGRCRMIRHS-RDKKNE 350

hB4GalT2 SKAQFLRINGFPNEYWGWGG**E**DDDIFNRIS-LTGMKIS**R**PDIRIGRYRMIKHD-RDKHNE 318

hB4GalT3 TPDQYLKMNGFPNEYWGWGG**E**DDDIATRVR-LAGMKIS**R**PPTSVGHYKMVKHR-GDKGNE 298

hB4GalT4 SREQFFKVNGFSNNYWGWGG**E**DDDLRLRVE-LQRMKIS**R**PLPEVGKYTMVFHT-RDKGNE 296

hB4GalT5 TVEQFRKINGFPNAFWGWGG**E**DDDLWNRVQ-NAGYSVS**R**PEGDTGKYKSIPHH-H-RGEV 335

hB4GalT6 TVEQFRKINGFPNAFWGWGG**E**DDDLWNRVH-YAGYNVT**R**PEGDLGKYKSIPHH-H-RGEV 329

hB4GalT7 SKQHYRLCNGMSNRFWGWGR**E**DDEFYRRIK-GAGLQLF**R**PSGITTGYKTFRHL-HDPAWR 264

hB4GalNAcT3 NTKEFRD---------RWGG**E**DWELLDRIL-QAGLDVE**R**LSLR-----NFFHHFHSKRGM 989

hB4GalNAcT4 NTEEFRD---------QWGG**E**DWELLDRVL-QAGLEVE**R**LRLR-----NFYHHYHSKRGM 1025

hCHSY1 DV-----------SIQGWGL**E**DVDLFNKVV-QAGLKTF**R**SQEV-----GVVHVHHPVFCD 752

hCHSY2 LAAA--------SEQEEELL**E**SLDVYELFLHFSSLHVL**R**AVEP-----ALLQRYRAQTCS 737

hCHGN1 DL-----------DIKGWGG**E**DVHLYRKYL-HSNLIVV**R**TPVR-----GLFHLWHEKRCM 482

hCHGN2 DM-----------EVKGWGG**E**DVHLYRKYL-HGDLIVI**R**TPVP-----GLFHLWHEKRCA 491

hCHPF1 DT-----------SIQGWGL**E**DVDLYNKVI-LSGLRPF**R**SQEV-----GVVHIFHPVHCD 839

hCHPF2 LAGEL------AGQEEEEAL**E**GLEVMDVFLRFSGLHLF**R**AVEP-----GLVQKFSLRDCS 726

hB4GalT1 PN--PQR**FD**R**IAH**T**K**---ETMLSDGLNSLTYQVLDVQRYPL------YTQITVDIGTPS- 398

hB4GalT2 PN--PQRFTKIQNTK---LTMKRDGIGSVRYQVLEVSRQPL------FTNITVDIGRPPS 367

hB4GalT3 EN--PHRFDLLVRTQ---NSWTQDGMNSLTYQLLARELGPL------YTNITADIGTDPR 347

hB4GalT4 VN--AERMKLLHQVS---RVWRTDGLSSCSYKLVSVEHNPL------YINITVDFWFGA- 344

hB4GalT5 QF--LGRYALLRKSK---ERQGLDGLNNLNYFA-NITYDAL------YKNITVNLTPELA 383

hB4GalT6 QF--LGRYKLLRYSK---ERQYIDGLNNLIYRP-KILVDRL------YTNISVNLMPELA 377

hB4GalT7 KR--DQKR-IAAQKQEQFKVDREGGLNTVKYHVASRTALSVGGAPCTVLNIMLDCDKTAT 321

hB4GalNAcT3 WS--RRQMKTL------------------------------------------------- 998

hB4GalNAcT4 WS--VRSRKGSRT-----------GAS--------------------------------- 1039

hCHSY1 PNLDPKQYKMCLGSKA----STYGSTQQLAEMWLEKNDP-S------YSK---------- 791

hCHSY2 ARLSEDLYHRCLQSVL----EGLGSRTQLAMLLFEQEQG-N------ST----------- 775

hCHGN1 DELTPEQYKMCMQSKA----MNEASHGQLGMLVFRHEIE-A------HLRK--------- 522

hCHGN2 DELTPEQYRMCIQSKA----MNEASHSHLGMLVFREEIE-T------HLHK--------- 531

hCHPF1 PNLDPKQYKMCLGSKA----NTFASTMQLAELWLEKHLG-V------RYN---------- 878

hCHPF2 PRLSEELYHRCRLSNL----EGLGGRAQLAMALFEQEQA-N------ST----------- 764

hB4GalT1 ----------------------------------------------- 398

hB4GalT2 -WPPRG----------------------------------------- 372

hB4GalT3 -GPRAPSGPRYPPGSSQAFRQEMLQRRPPARPGPLSTANHTALRGSH 393

hB4GalT4 ----------------------------------------------- 344

hB4GalT5 -QV-----NEY------------------------------------ 388

hB4GalT6 -PI-----EDY------------------------------------ 382

hB4GalT7 PWCT------FS----------------------------------- 327

hB4GalNAcT3 ----------------------------------------------- 998

hB4GalNAcT4 ----------------------------------------------- 1039

hCHSY1 -----------SSNNNGSVRTA------------------------- 802

hCHSY2 ----------------------------------------------- 775

hCHGN1 --------QKQKTSSKKT----------------------------- 532

hCHGN2 --------QAYRTNSEAVG---------------------------- 542

hCHPF1 -----------RTLS-------------------------------- 882

hCHPF2 ----------------------------------------------- 764
