# Supplementary material for: The dimeric structure of wild-type human glycosyltransferase B4GalT1
Source: PLoS One. 2018 Oct 23;13(10):e0205571. doi: 10.1371/journal.pone.0205571 (PMC6198961; doi:10.1371/journal.pone.0205571)
Supplement: S7 Fig — (DOCX) [file pone.0205571.s011.docx]

**S7 Fig. Multiple sequence alignment of the B4GalT1 protein among animal species**

CLUSTAL O (1.2.4) multiple sequence alignment of the B4GalT1 protein among animal species. The dimerization interface, as observed in the closed structure of B4GalT1, is indicated as bold on yellow background. Residues E313 and R330 of the hB4GalT’s and the corresponding residues appearing in the sequences of the other GT7 family enzymes are given as bold and red. The Trp loop is marked with turquoise background.

The names, sequences and organism (beginning with the gene name identifier) in the comparison are in the following order:

P15291 B4GT1_HUMAN Beta-1,4-galactosyltransferase 1 Homo sapiens

P08037 B4GT1_BOVIN Beta-1,4-galactosyltransferase 1 Bos taurus

P15535 B4GT1_MOUSE Beta-1,4-galactosyltransferase 1 Mus musculus

Q92074 Q92074_CHICK Beta-1,4-galactosyltransferase 1 Gallus gallus

K7BHK3 K7BHK3_PANTR B4GALT1 isoform 1 Pan troglodytes

G1TZ23 G1TZ23_RABIT Beta-1,4-galactosyltransferase 1 Oryctolagus cuniculus

G3R175 G3R175_GORGO Beta-1,4-galactosyltransferase 1 Gorilla gorilla gorilla

I3M5Q2 I3M5Q2_ICTTR Beta-1,4-galactosyltransferase 1 Ictidomys tridecemlineatus

H0WPR4 H0WPR4_OTOGA Beta-1,4-galactosyltransferase 1 Otolemur garnettii

A0A287 G16|A0A287BG16_PIG Beta-1,4-galactosyltransferase 1 Sus scrofa

M3XYN6 M3XYN6_MUSPF Beta-1,4-galactosyltransferase 1 Mustela putorius furo

G1LLX7 G1LLX7_AILME Beta-1,4-galactosyltransferase 1 Ailuropoda melanoleuca

M3WTX2 M3WTX2_FELCA Beta-1,4-galactosyltransferase 1 Felis catus

W5PIH2 W5PIH2_SHEEP Beta-1,4-galactosyltransferase 1 Ovis aries

F7I1Z8 F7I1Z8_CALJA Beta-1,4-galactosyltransferase 1 Callithrix jacchus

E9NRZ3 E9NRZ3_CAPHI Beta-1,4-galactosyltransferase I Capra hircus

G3V722 G3V722_RAT Beta-1,4-galactosyltransferase 1 Rattus norvegicus

F1PGZ1 F1PGZ1_CANLF Beta-1,4-galactosyltransferase 1 Canis lupus familiaris

F6SRF3 F6SRF3_HORSE Beta-1,4-galactosyltransferase 1 Equus caballus

Homo_sapiens MRLREPLLSGS-AAMPGASLQRACRLLVAVCALHLGVTLVYYLAGRDLSRLPQLVGVSTP 59

Bos_taurus MKFREPLLGGS-AAMPGASLQRACRLLVAVCALHLGVTLVYYLAGRDLRRLPQLVGVHPP 59

Mus_musculus MRFREQFLGGS-AAMPGATLQRACRLLVAVCALHLGVTLVYYLSGRDLSRLPQLVGVSST 59

Gallus_gallus ---------MKEPALPGTSLQRACRLLVAFCALHLSATLLYYLAGSSLTPPRSPEP---- 47

Pan_troglodytes MRLREPLLSGS-AAMPGASLQRACRLLVAVCALHLGVTLVYYLAGRDLSRLPQLVGVSTA 59

Oryctolagus_cuniculus MRFREPLLGGS-TAMPGASLQRACRLLVAVCALHLGVTLVYYLAGRDLSRLPQLVGVATQ 59

Gorilla_gorilla MRLREPLLSGS-AAMPGASLQRACRLLVAVCALHLGVTLVYYLAGRDLSRLPQLVGVSTP 59

Ictidomys_tridecemlineatus MRFREPLLGGS-AAMPGASLQRACRLLVAVCALHLGVTLVYYLAGRDLSRLPQLVGVSTS 59

Otolemur_garnettii MRFREPLLGGS-AAMPGASLQRACRLLVAVCALHLGVTLVYYLAGRDLSRLPQLVGVSTS 59

Sus_scrofa MRFREPLLGGSAATMPGASLQRACRLLVAVCALHLGVTLIYYLTGRDLSRLPQLVGVPTP 60

Mustela_putorius --------------MPGASLQRACRLLVAVCALHLGGTLVYYLAGRDLSRLPQLVGVPTP 46

Ailuropoda_melanoleuca RRWRKRRRRGSVATMPGASLQRACRLLVFVCALHLGVTLVYYLAGRDLSRLPQLVGVPTP 60

Felis_catus --------------MPGASLQRACRLLVAVCALHLGVTLVYYLAGRDLNRLPQLVGVPTP 46

Ovis_aries MRFREPLLGGS-AAMPGASLQRACRLLVAVCALHLGVTLVYYLAGRDLRRLPQLVGVHTT 59

Callithrix_jacchus MRFREPLLGGS-AAMPGASLQRACRLLVAVCALHLGVTLVYYLAGRDLRRLPQLVGVSTP 59

Capra_hircus MKFREPLLGGS-AAMPGASLQRACRLLVAVCALHLGVTLVYYLAGRDLRRLPQLVGVRTT 59

Rattus_norvegicus MRFREPFLGGS-AAMPGATLQRACRLLVAVCALHLGVTLVYYLSGRDLSRLPQLVGVSSS 59

Canis_lupus ------------------------------------------------------------ 0

Equus_caballus ------------------------------------------------------------ 0

Homo_sapiens LQGGSNSAA--AIGQSSGELRTGGARPPPPLGASSQPRPGGDSSPVVDSG----PGPASN 113

Bos_taurus LQGSSHGAA--AIGQPSGELRLRGVAPPPPLQNSSKPRSRAPSNLDAYSHPGPGPGPGSN 117

Mus_musculus LQGGTNGAA--ASKQPPGEQRPRGARPPPPLGVSPKPRPGLDSSPGAASG----PGLKSN 113

Gallus_gallus ----------PPRRPPPANLSLPPSRPPPP--------------PAARPRPGPVSAQPRN 83

Pan_troglodytes LQGGSNGAA--ANGQSSGELRTGGARPPPPLGASSQPRPGGDSSPVVDSG----PGPASN 113

Oryctolagus_cuniculus LQGGSSGATAASSGQPSGELQAGGVRPPPPLGTFSEPRRGHDSSPGADSG----PGPASN 115

Gorilla_gorilla LQGGSNGAA--AIGQSSGELRTGGARPPPPLGASSQPRPGGDSSPVVDSG----PGPASN 113

Ictidomys_tridecemlineatus LQGGSNGAT--SNGQTSGELRPGGARPPPPLGVSSEPRPGLDSSPGANSG----PALASN 113

Otolemur_garnettii LQGGSHGTA--AIGQPSGDIRSRGVRPPPPLGASPEPRPGRDSSPHADSG----RGLASN 113

Sus_scrofa LQGGSNSAA--AIGQPSGELRPRGAAPQPPLRASSKPSSGGDSSPDADSR--PGPGPASN 116

Mustela_putorius LQGGSNGAA--AIEQPNGELRPGGAPPLPPSDASSELRSGRGSSPSTDSH--PGPGSASN 102

Ailuropoda_melanoleuca LQGGSNGAA--AIEQPSGELRPRGAPPLPPLDASSELRSGHDSSPDTDSH--PGPGS--N 114

Felis_catus LQGGSNGAA--AIEQPSAELRPRGAPPLPLLDASSELRSGRDSSPDADSH--PGPGPASN 102

Ovis_aries LQGSSHGAA--AIGQPSGELRLRGAAPPPPLQNSSELRSRAASNPDADSRPGPGPDPGSN 117

Callithrix_jacchus LQGGSNGAA--AIGQPSGELRARGVRPPPPLGASSQPRPGGDSSPVADSG----PGPANN 113

Capra_hircus LQGSSHGAA--AIGQPSGELRLRGAAPPPPLQNSSELRSRAASNPDADSRPGPGPGPGSN 117

Rattus_norvegicus LQGGTNGAA--ASKQPSGELRPRGARPPPPLGVSPKPRPGSDSSPDAASG----PGLKSN 113

Canis_lupus ------------------------------------------------------------ 0

Equus_caballus ------------------------------------------------------------ 0

Homo_sapiens LTSVPVPHTTAL--SLPACPEESPLLVGPMLIEFNMPVDLELVAKQNPNVKMGGRYAPRD 171

Bos_taurus LTSAPVPSTTT--RSLTACPEESPLLVGPMLIEFNIPVDLKLVEQQNPKVKLGGRYTPMD 175

Mus_musculus LSSLPVPTTTGL-LSLPACPEESPLLVGPMLIDFNIAVDLELLAKKNPEIKTGGRYSPKD 172

Gallus_gallus L---------PDSAPSGLCPDPSPLLVGPLRVEFSQPVNLEEVASTNPEVREGGRFAPKD 134

Pan_troglodytes LTSVPVPHTTAL--SLPACPEESPLLVGPMLIEFNMPVDLELVAKQNPNVKMGGRYAPRD 171

Oryctolagus_cuniculus LTSVPVLPTTA--LLLPVCPEESPLLVGPMLIEFNIPVDLELLAKQNPEVKVGGRYTPKD 173

Gorilla_gorilla LTSVPVPHTTAL--SLPACPEESPLLVGPMLIEFNKPVDLELVAKQNPNVKMGGRYAPRD 171

Ictidomys_tridecemlineatus LTSALVSSTSA--LSLLACPEESPLLVGPMLIEFNIPVDLELLAKKNPEVKVGGRYTPKD 171

Otolemur_garnettii LTSVPVSRTTK--LPLPACPEESPLLVGPMLIEFNMPVDLELVAKMNPKVKIGGRYTPKE 171

Sus_scrofa LTSAPVPSTAA--RLPPACPEESPLLVGPMLIEFNMAVDLKLVEKQNPEVNMGGHYTPKD 174

Mustela_putorius LTSAPVSFTSA--LSLLACPEESPLLVGPMVIEFNMPVDLKLVEKQNPEVKVGGRYTPKN 160

Ailuropoda_melanoleuca LTSAPVPSTTA--LSLLACPEESPLLVGPMVIEFNMPVDLKLVEKQNPEVKVGGRYTPKN 172

Felis_catus LTSAPVPSTTV--LSLLACPEESPLLVGPMVIEFNMPVDLKLVEKQNPEVKVGGRYTPKN 160

Ovis_aries LTSAPVPSTTT--LSLTECPEESPLLVGPMLIEFNIPVDLKLVEQQNPKVKLGGRYSPTD 175

Callithrix_jacchus LTSVPVPRTTALALPLPACPEESPLLVGPMLIEFNMPVDLELVAKQNPNVKMGGRYTPKD 173

Capra_hircus LTSAPVPSTTT--LSLTECPEESPLLVGPMLIEFNIPVDLKLVEQQNPKVKLGGRYSPTD 175

Rattus_norvegicus LTSVPMPTSTGL-LTLPACPEESPLLVGPMVIDFNIPVDLELLAKKNPEIKMGGRYFPKD 172

Canis_lupus -----------------------------MVIEFNMPVDLKLVEKQNPEVKVGGRYTPKN 31

Equus_caballus ---------------------SSTSLVGPMMIEFNMAVDLNRVAEENPEVKLGGRYTPKD 39

Homo_sapiens CVSPHKVAIIIPFRNRQEHLKYWLYYLHPVLQRQQLDYGIYVINQAGDTIFNRAKLLNVG 231

Bos_taurus CISPHKVAIIIPFRNRQEHLKYWLYYLHPILQRQQLDYGIYVINQAGESMFNRAKLLNVG 235

Mus_musculus CVSPHKVAIIIPFRNRQEHLKYWLYYLHPILQRQQLDYGIYVINQAGDTMFNRAKLLNIG 232

Gallus_gallus CKALQKVAIIIPFRNREEHLKYWLYYMHPILQRQQLDYGVYVINQDGDEEFNRAKLLNVG 194

Pan_troglodytes CVSPHKVAIIIPFRNRQEHLKYWLYYLHPVLQRQQLDYGIYVINQAGDTMFNRAKLLNVG 231

Oryctolagus_cuniculus CISPHKVAIIIPFRNRQEHLKYWLYYLHPILQRQQLDYGIYVINQAGDSMFNRAKLLNIG 233

Gorilla_gorilla CVSPHKVAIIIPFRNRQEHLKYWLYYLHPVLQRQQLDYGIYVINQAGDTMFNRAKLLNVG 231

Ictidomys_tridecemlineatus CISPHKVAIIIPFRNRQEHLKYWLYYLHPILQRQQLDYGIYVINQAGDTMFNRAKLLNIG 231

Otolemur_garnettii CVSPHKVAIIIPFRNRQEHLKYWLYYLHPILQRQQLDYGIYVINQAGDSKFNRAKLLNVG 231

Sus_scrofa CTSPHKVAIIIPFRNRQEHLKYWLYYLHPILQRQQLDYGVYVINQAGESMFNRAKLLNVG 234

Mustela_putorius CISPHKVAIVIPFRNRQEHLKYWLYYLHPILQRQQLDYGIYVINQAGETMFNRAKLLNIG 220

Ailuropoda_melanoleuca CISPHKVAIIIPFRNRQEHLKYWLYYLHPVLQRQQLDYGIYVVNQAGETMFNRAKLLNIG 232

Felis_catus CISPHKVAIIIPFRNRQEHLKYWLYYLHPILQRQQLDYGIYVINQAGETMFNRAKLLNIG 220

Ovis_aries CISPHKVAIIIPFRNRQEHLKYWLYYLHPILQRQQLDYGIYVINQAGESMFNRAKLLNVG 235

Callithrix_jacchus CISPHKVAIIIPFRNRQEHLKYWLYYLHPVLQRQQLNYGIYVINQAGDTMFNRAKLLNVG 233

Capra_hircus CISPHKVAIIIPFRNRQEHLKYWLYYLHPILQRQQLDYGIYVINQAGESMFNRAKLLNVG 235

Rattus_norvegicus CISPHKVAIIIPFRNRQEHLKYWLYYLHPVLQRQQLDYGIYVINQAGDTMFNRAKLLNVG 232

Canis_lupus CISPHKVAIIIPFRNRQEHLKYWLYYLHPILQRQQLDYGIYVINQAGETMFNRAKLLNIG 91

Equus_caballus CISPHKVAIIIPFRNRQEHLKYWLYYLHPILQRQQLDYGIYVINQAGEAMFNRAKLLNVG 99

Homo_sapiens FQEALKDYDYTCFVFSDVDLIPMNDHNAYRCF**SQ**PRHIS**VA**MDK**FGFSLP**YVQ**Y**FGGVSA 291

Bos_taurus FKEALKDYDYNCFVFSDVDLIPMNDHNTYRCFSQPRHISVAMDKFGFSLPYVQYFGGVSA 295

Mus_musculus FQEALKDYDYNCFVFSDVDLIPMDDRNAYRCFSQPRHISVAMDKFGFSLPYVQYFGGVSA 292

Gallus_gallus FTEALKEYDYDCFVFSDVDLIPMDDRNTYKCYSQPRHLSVSMDKFGFRLPYNQYFGGVSA 254

Pan_troglodytes FQEALKDYDYTCFVFSDVDLIPMNDHNAYRCFSQPRHISVAMDKFGFSLPYVQYFGGVSA 291

Oryctolagus_cuniculus FQEALKDYDYNCFVFSDVDLIPMDDHNAYRCFSQPRHISVAMDKFGFSLPYVQYFGGVSA 293

Gorilla_gorilla FQEALKDYDYTCFVFSDVDLIPMNDHNAYRCFSQPRHISVAMDKFGFSLPYVQYFGGVSA 291

Ictidomys_tridecemlineatus FQEALKDYDYNCFVFSDVDLIPMDDHNAYRCFPQPRHISVAMDKFGFSLPYVQYFGGVSA 291

Otolemur_garnettii FQEALKDYDYNCFVFSDVDLIPMDDHNAYRCFSQPRHISVAMDKFGFSLPYVQYFGGVSA 291

Sus_scrofa FKEALKDYDYNCFVFSDVDLIPMNDHNAYRCFSQPRHISVAMDKFGFSLPYVQYFGGVSA 294

Mustela_putorius FQEALKDYDYNCFVFSDVDLIPMNDRNAYRCFPQPRHISVAMDKFGFSLPYVQYFGGVSA 280

Ailuropoda_melanoleuca FQEALKDYDYNCFVFSDVDLIPMNDHNAYRCFSQPRHISVAMDKFGFSLPYVQYFGGVSA 292

Felis_catus FQEALKDYDYNCFVFSDVDLIPMNDHNAYRCFSQPRHISVAMDKFGFR------------ 268

Ovis_aries FKEALKDYDYNCFVFSDVDLIPMNDHNTYRCFSQPRHISVAMDKFGFSLPYVQYFGGVSA 295

Callithrix_jacchus FREALKDYDYTCFVFSDVDLIPMDDHNAYRCFSQPRHISVAMDKFGFSLPYVQYFGGVSA 293

Capra_hircus FKEALKDYDYNCFVFSDVDLIPMNDHNTYRCFSQPRHISVAMDKFGFSLPYVQYFGGVSA 295

Rattus_norvegicus FQEALKDYDYNCFVFSDVDLIPMDDHNAYRCFSQPRHISVAMDKFGFSLPYVQYFGGVSA 292

Canis_lupus FQEALKDYDYNCFVFSDVDLIPMNDHNAYRCFSQPRHISVAMDKFGFSLPYVQYFGGVSA 151

Equus_caballus FQEALKDYDYNCFVFSDVDLIPMNDHNAYRCFSQPRHISVAMDKFGFSLPYVQYFGGVSA 159

Homo_sapiens LSKQQFLTINGFPNNYWGWGG**E**DDDI**FN**RL**VFRGMSISR**PN**A**VVGRCRMIRHSRDKKNEP 351

Bos_taurus LSKQQFLSINGFPNNYWGWGG**E**DDDIYNRLAFRGMSVS**R**PNAVIGKCRMIRHSRDKKNEP 355

Mus_musculus LSKQQFLAINGFPNNYWGWGG**E**DDDIFNRLVHKGMSIS**R**PNAVVGRCRMIRHSRDKKNEP 352

Gallus_gallus LSKEQFTKINGFPNNYWGWGG**E**DDDIYNRLVFKGMGIS**R**PDAVIGKCRMIRHSRDRKNEP 314

Pan_troglodytes LSKQQFLTINGFPNNYWGWGG**E**DDDIFNRLVFRGMSIS**R**PNAVVGRCRMIRHSRDKKNEP 351

Oryctolagus_cuniculus LSKDQFLAINGFPNNYWGWGG**E**DDDIFNRLVFRGMSIS**R**PNAVVGRCRMIRHSRDKKNEP 353

Gorilla_gorilla LSKQQFLTINGFPNNYWGWGG**E**DDDIFNRLVFRGMSIS**R**PNAVVGRCRMIRHSRDKKNEP 351

Ictidomys_tridecemlineatus LSKQQFLTINGFPNNYWGWGG**E**DDDIFNRLVFRGMSIS**R**PNAVIGRCRMIRHSRDKKNEP 351

Otolemur_garnettii LSKQQFLKINGFPNNYWGWGG**E**DDDIFNRLVFKGMSIS**R**PNAVVGRCRMIRHSRDKKNEP 351

Sus_scrofa LSKEQFLTINGFPNNYWGWGG**E**DDDIFNRLAFKGMSVS**R**PNAMIGKCRMIRHSRDKKNEP 354

Mustela_putorius LSKQQFLTINGFPNNYWGWGG**E**DDDIFNRLVFRGMSVS**R**PNAVVGKCRMIRHSRDKKNEP 340

Ailuropoda_melanoleuca LSKQQFLTINGFPNNYWGWGG**E**DDDIFNRLVFKGMSVS**R**PNAVVGKCRMIRHSRDKKNEP 352

Felis_catus -----------------------------LVFRGMSVS**R**PNAVVGKCRMIRHSRDKKNEP 299

Ovis_aries LSKQQFLSINGFPNNYWGWGG**E**DDDIYNRLAFRGMSVS**R**PNAVIGKCRMIRHLRDKKNEP 355

Callithrix_jacchus LSKQQFLTINGFPNNYWGWGG**E**DDDIFNRLVFKGMSIS**R**PNAVVGRCRMIRHSRDKKNEP 353

Capra_hircus LSKQQFLSINGFPNNYWGWGG**E**DDDIYNRLAFRGMSVS**R**PNAVIGKCRMIRHSRDKKNEP 355

Rattus_norvegicus LSKQQFLTINGFPNNYWGWGG**E**DDDIFNRLVHKGMSIS**R**PNAVVGRCRMIRHSRDKKNEP 352

Canis_lupus LSKEQFLTINGFPNNYWGWGG**E**DDDIYNRLVFKGMSVS**R**PNAMVGKCRMIRHSRDKKNEP 211

Equus_caballus LSKEQFLTINGFPNNYWGWGG**E**DDDIFNRLVFKGMSLS**R**PNAVIGKCRMIRHSRDKKNEP 219

Homo_sapiens NPQR**FD**R**IAH**T**K**ETMLSDGLNSLTYQVLDVQRYPLYTQITVDIGTPS- 398

Bos_taurus NPQRFDRIAHTKETMLSDGLNSLTYMVLEVQRYPLYTKITVDIGTPS- 402

Mus_musculus NPQRFDRIAHTKETMRFDGLNSLTYKVLDVQRYPLYTQITVDIGTPR- 399

Gallus_gallus NPERFDRIAHTRETMSSDGLNSLSYEVLRTDRFPLYTRITVDIGAPGS 362

Pan_troglodytes NPQRFDRIAHTKETMLSDGLNSLTYQVLDVQRYPLYTQITVDIGTPS- 398

Oryctolagus_cuniculus NPQRFDRIAHTKETMRSDGLNSLTYQVLDIQRNPLYTKITVDIGTPS- 400

Gorilla_gorilla NPQRFDRIAHTKETMLSDGLNSLTYQVLDVQRYPLYTQITVDVGTPS- 398

Ictidomys_tridecemlineatus NPQRFDRIAHTRDTMRFDGLNSLTYHVLDVQRYPLYTKITVDIGTPS- 398

Otolemur_garnettii NPQRFDRIAHTKETMLSDGLNSLTYELLDVQRYPMYTQITVDVGTPS- 398

Sus_scrofa NPQRFDRIAHTKETMLSDGLNTLTYLVLDIERYPLFTKITVDIGTPS- 401

Mustela_putorius NPQRFDRIAHTKETMLSDGLNTLTYKVLDIERNPLYTKITVDIGTPS- 387

Ailuropoda_melanoleuca NPQRFDRIAHTKETMLLDGLNTLTYKVLDIERNPLYTKITVDIGTPS- 399

Felis_catus NPQRFDRIAHTKETMLSDGLNTLSYKVLDIERNPLYTKITVDIGTPS- 346

Ovis_aries NPQRFDRIAHTKETMLSDGLNSLTYMVLEVQRYPLYTKITVDIGTPS- 402

Callithrix_jacchus NPQRFDRIAHTKETMLSDGLNSLTYQVLDVQRYPLYTQITVDIGTPS- 400

Capra_hircus NPQRFDRIAHTKETMLSDGLNSLTYMVLEVQRYPLYTKITVDIGTPS- 402

Rattus_norvegicus NPQRFDRIAHTKETMRLDGLNSLTYQVLDIQRYPLYTKITVDIGTPR- 399

Canis_lupus NPQRFDRIAHTKETMLSDGLNTLTYKVLDKERNPLYTKITVDIGTPS- 258

Equus_caballus NPQRFDRIAHTKETMFLDGLNTLFYNVLDVQRYPLYTKVTVDIGTPS- 266
